# A tailored double perovskite nanofiber catalyst enables ultrafast oxygen evolution

**DOI:** 10.1038/ncomms14586

**Published:** 2017-02-27

**Authors:** Bote Zhao, Lei Zhang, Dongxing Zhen, Seonyoung Yoo, Yong Ding, Dongchang Chen, Yu Chen, Qiaobao Zhang, Brian Doyle, Xunhui Xiong, Meilin Liu

**Affiliations:** 1 School of Materials Science and Engineering, Georgia Institute of Technology, Atlanta, Georgia 30332-0245, USA; 2 New Energy Research Institute, School of Environment and Energy, South China University of Technology, Guangzhou 510006, China

## Abstract

Rechargeable metal–air batteries and water splitting are highly competitive options for a sustainable energy future, but their commercialization is hindered by the absence of cost-effective, highly efficient and stable catalysts for the oxygen evolution reaction. Here we report the rational design and synthesis of a double perovskite PrBa_0.5_Sr_0.5_Co_1.5_Fe_0.5_O_5+*δ*_ nanofiber as a highly efficient and robust catalyst for the oxygen evolution reaction. Co-doping of strontium and iron into PrBaCo_2_O_5+*δ*_ is found to be very effective in enhancing intrinsic activity (normalized by the geometrical surface area, ∼4.7 times), as validated by electrochemical measurements and first-principles calculations. Further, the nanofiber morphology enhances its mass activity remarkably (by ∼20 times) as the diameter is reduced to ∼20 nm, attributed to the increased surface area and an unexpected intrinsic activity enhancement due possibly to a favourable e_g_ electron filling associated with partial surface reduction, as unravelled from chemical titration and electron energy-loss spectroscopy.

## Abstract (web summary)

The design of efficient and stable oxygen evolution catalysts has implications for water splitting and metal-air battery technology. Here, the authors fabricate double perovskite nanofibers and demonstrate the favourable effects of co-doping and nanostructuring on oxygen evolution performance.

The oxygen evolution reaction (OER), an essential but sluggish step in many energy storage and conversion processes, has received significant attention, particularly in the development of solar/electricity-driven water splitting and rechargeable metal–air batteries^1^,^2^,^3^,^4^,^5^,^6^. To date, however, a large overpotential (*η*) is still required to accelerate the rate of the multistep electron transfer processes to deliver a desirable current density. The development of highly efficient OER catalysts is critical to achieving fast kinetics. Precious metal oxides (for example, RuO_2_ and IrO_2_) have demonstrated reasonably high OER activity. In particular, IrO_2_ has been considered the state-of-the-art electrocatalyst^1^. However, the high cost prohibits their widespread use in practical applications.

As an alternative cost-effective catalyst with high intrinsic OER activities, perovskite oxides (ABO_3_, where A is a rare earth or alkaline earth metal ion and B is a transition metal ion) are of particular interest^7^. The substitution of A and/or B cations by ions of different radii, valencies and electronegativities has been successfully used to tailor the physical, chemical and catalytic properties. To date, considerable efforts have been devoted to understanding the OER mechanism of perovskite catalysts, identifying reliable descriptors of OER activity, and developing highly efficient perovskite catalysts^8^. On the basis of systematic experiments and molecular orbital principles, the e_g_ occupancy of surface cations has been proposed as a dependable descriptor for OER activity; Ba_0.5_Sr_0.5_Co_0.8_Fe_0.2_O_3−*δ*_ (BSCF) with an optimal e_g_≈1.2 was identified with a landmark intrinsic OER activity, about one order of magnitude higher than that of IrO_2_ catalyst^1^. Unfortunately, the surface amorphization with formation of structural motifs with local order of edge-sharing octahedra was found for BSCF during the OER^9^,^10^. Strategies such as A/B cation substitution (or doping)^11^,^12^,^13^,^14^, nanostructure engineering^11^,^15^,^16^ and surface treatment^17^,^18^ have been successfully applied to enhance activity and/or stability. Double perovskites (AA′B_2_O_5+*δ*_) were found to have stable structure during the OER due to proper O *p*-band centre position relative to the Fermi level^19^; in particular, PrBaCo_2_O_5+*δ*_ (PBC) was identified with optimal intrinsic OER activity, comparable with that of BSCF catalyst^19^. The intrinsic activity, also known as specific activity that is defined as the surface area-normalized kinetic current density (where the surface area can be measured electrochemically^20^ or geometrically^1^,^19^,^21^), is essential to characterizing the optimum catalytic behaviour for OER. In addition to the intrinsic activity, the mass activity of the catalyst will be more crucial to the practical application. Unfortunately, a high calcination temperature, especially for double perovskites^19^,^22^,^23^,^24^, is obligatory for the preparation of phase-pure perovskite oxides, resulting in a significantly lower surface area and thus low mass activity. Therefore, development of perovskite catalysts for OER with combined properties of high mass activity, intrinsic activity as well as durability remains a critical challenge.

In this work, we report our findings on the rational design, controlled synthesis and characterization of double perovskite PrBa_0.5_Sr_0.5_Co_1.5_Fe_0.5_O_5+*δ*_ (PBSCF) as a highly efficient and stable catalyst for OER, attributed to both co-doping and nanostructure engineering. A PBSCF nanofiber of ∼20 nm in diameter achieves ∼72-fold enhancement in mass activity at an overpotential of 0.37 V compared with PBC powders. Our rational design of the catalyst includes co-doping of Sr and Fe into PBC perovskite oxide to enhance OER intrinsic activity and tailoring the nanostructure of PBSCF fibers to further enhance its mass activity by increasing the surface area. Co-doping is first applied to PBC for enhancing the intrinsic or specific OER activity (that is, the Brunauer−Emmett−Teller (BET) surface area-normalized kinetic current density in this work), which is validated by electrochemical measurements and first-principles calculations. We further demonstrate the controlled synthesis of PBSCF nanofibers with diameters from ∼196 to 20 nm to enhance the OER mass activity and understand the nanosize effect. Importantly, PBSCF nanofibers of ∼20 nm in diameter show markedly enhanced OER activities compared to that of the PBSCF powders, superior to the commercial IrO_2_ catalyst and those of recently reported advanced perovskite catalysts^11^,^12^,^13^,^14^,^15^,^17^,^18^. An enhancement in intrinsic activity of the same PBSCF material with decreasing the diameter to ∼20 nm (ultrafine nanofiber versus micron-sized powder) is observed, which is explained by favourable e_g_ electron filling of ultrafine nanofiber, stronger adsorption of oxygen-containing adsorbates, possible surface reduction and heterostructure. On the basis of its superior activity and stability, the ultrafine PBSCF nanofiber has potential to be a very promising candidate of the next-generation electrocatalysts for OER. This work not only represents an advancement in the development of highly efficient and durable electrocatalysts for OER but may also provide insight into the effect of nanostructures on the intrinsic OER activity.

## Results

### Structure and microstructure characterizations

**Figure 1:**
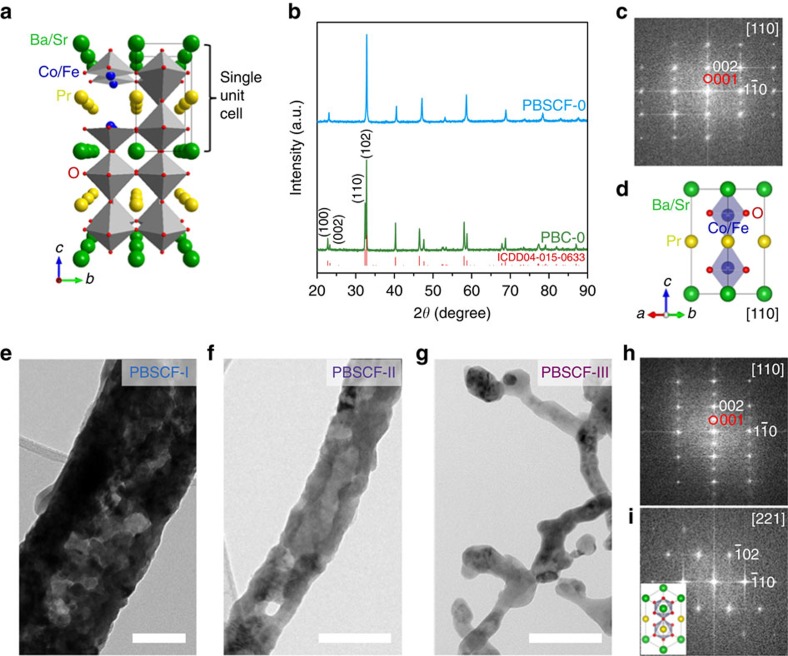
Structure and morphology characterization. (**a**) Schematic of PrBaCo_2_O_5+*δ*_ (PBC)/PrBa_0.5_Sr_0.5_Co_1.5_Fe_0.5_O_5+*δ*_ (PBSCF) double perovskite crystal structure. (**b**) XRD patterns of PBC-0 and PBSCF-0 powders. (**c**) Fast Fourier transform (FFT) pattern (obtained from region of ∼23 nm × 23 nm) and (**d**) corresponding schematic crystal structure of single unit cell of PBSCF. (**e**–**g**) Bright-field TEM images of (**e**) PBSCF-I, (**f**) PBSCF-II and (**g**) PBSCF-III nanofibers. (**h**,**i**) FFT patterns (obtained from region of ∼19 nm × 19 nm and ∼15 nm × 15 nm, respectively) of PBSCF-III; inset in **i** is the schematic crystal structure of single unit cell of PBSCF along [221] zone axis. Red circles indicate the superlattice reflections, which are characteristics of the double perovskite structure. Scale bar, 100 nm (**e**–**g**).

Double perovskite PBC has an ideal layered structure (Fig. 1a) with Pr and Ba cations ordered in alternating layers along the *c* axis, that is, [BaO]–[CoO_2_]–[PrO_*δ*_]–[CoO_2_]–[BaO], where the oxygen vacancies are confined only to [PrO_δ_] layer^22^,^25^. By co-doping of Sr and Fe into PBC, ideally, Sr replaces Ba, and Fe occupies B site (Co), respectively (Fig. 1a). Since the ionic radius of Sr^2+^ (1.44 Å in 12-fold coordination) is not much larger than that of Pr^3+^ (∼1.31 Å, extrapolated to 12-fold coordination) compared to Ba^2+^ (1.61 Å in 12-fold coordination)^26^, the structure will change somewhat by excess Sr-doping. When Ba is fully replaced by Sr, the structure changed to a simple perovskite^27^. To enhance the electrochemical performance while maintaining the structure, half of the Ba atoms are usually substituted by Sr (refs 23, 27, 28). It was demonstrated that Sr- or Fe-doping will change the electrical conductivity, oxygen content and thermal expansion coefficient of PBC-based cathodes for solid oxide fuel cells (SOFCs)^27^,^29^. A synergistic effect of Sr and Fe co-doping in PBC (that is, PBSCF) for SOFC has been reported by creating pore channels that markedly enhanced oxygen ion diffusion and surface oxygen exchange^24^. However, the electrode processes on a cathode of SOFC at high temperatures are very different from those on an OER catalyst in a liquid electrolyte at room temperature.

To understand the effect of co-doping on OER activity, we synthesized PBC and PBSCF powders (denoted as PBC-0 and PBSCF-0, respectively). The X-ray diffraction patterns shown in Fig. 1b suggest that pure double perovskite phases (ICDD04-015-0633) are obtained. The obvious splitting of the peaks correponded to (100)/(002) and (110)/(102) diffracttion planes of PBC-0 is characteristic for double perovskite phase. These two pairs of peaks were overlapped for PBSCF-0, suggesting that the lattice parameters *c* is very close to 2*a*_*p*_ (the cell parameter of the ideal cubic perovskite), which is consistent with the literature^24^. The morphologies of these powders were similar with particle diameters of ∼0.4−2.5 μm (Supplementary Fig. 1). The fast Fourier transform pattern along [110] zone axis (Fig. 1c) shows the presence of superlattice reflections along the *c* axis (Fig. 1d), confirming the successful formation of double perovskite structure for PBSCF-0. However, the superlattice reflection dots became weak in some selected regions, suggesting that the double perovskite structure is somewhat disordered mainly because the ionic radius of Sr^2+^ is comparable to that of Pr^3+^.

To increase the surface area for enhanced OER mass activity, PBSCF nanofibers with small diameters were fabricated by a controlled electrospinning process. PBSCF precursor nanofibers with three different diameters were designed and synthesized (Supplementary Fig. 1; Supplementary Table 1). To maximize the surface area and thus the OER mass activity, a low calcination temperature of 750 °C was used for all PBSCF nanofibers to ensure that the finest PBSCF nanofiber (denoted as PBSCF-III) had a pure phase (Supplementary Fig. 2). This temperature was significantly lower than that for other synthesis methods (Supplementary Note 1). Although the PBSCF-III sample calcined at 750 °C was somewhat sintered, they still appear to be nanofibers. The PBSCF nanofibers with mean diameters of 196, 83 and 20 nm were obtained after calcination and denoted as PBSCF-I, PBSCF-II and PBSCF-III, respectively (Supplementary Figs 1,3 and 4). The representative bright-field transmission electron microscope (TEM) images of PBSCF-I, II and III nanofibers were shown in Fig. 1e,f,g, respectively. The PBSCF-I and PBSCF-II nanofibers have discontinuous pores inside (Fig. 1e; Supplementary Fig. 5a) and hollow structure with continuous mesopores (Fig. 1f; Supplementary Fig. 5b), respectively, which were created by outward diffusion of the gases from oxidization of polyvinylpyrrolidone (PVP) and decomposition of metal precursors. By further decreasing the diameter to ∼20 nm, the ultrafine PBSCF-III nanofibers were solid with no observable pores, and some abutting nanofibers were sintered together (Fig. 1g). The PBSCF-III was composed of sintered small grains, where the diameter of the nanofiber is the grain size (Supplementary Fig. 5c). It suggests that the diameter almost reaches the minimum limit at this calcination temperature. To the best of our knowledge, this is the finest diameter for reported electrospun perovskite oxide nanofibers. The double perovskite structure of PBSCF-III nanofiber with superstructure was confirmed by fast Fourier transform patterns along [110] and [221] zone axes (Fig. 1h,i), even though the structure is not in perfectly layered structure similar to the case of PBSCF-0. The BET surface areas of the PBSCF samples were successfully increased with decreasing the particle sizes (Supplementary Fig. 6; Supplementary Table 2), that is, 1.52, 9.09, 14.72 and 18.81 m^2^ g^−1^ for PBSCF-0, I, II and III, respectively.

### Characterization of the electrochemical performance

**Figure 2:**
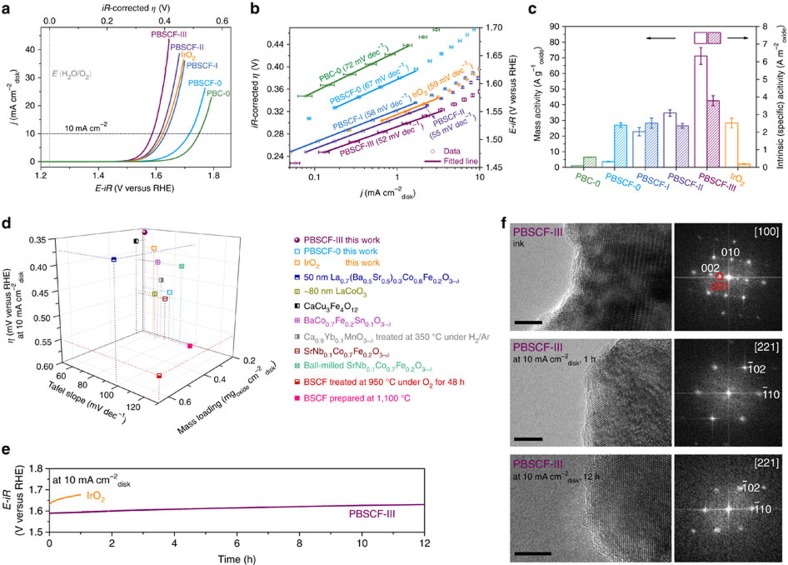
Electrochemical properties and structural stability of catalysts. (**a**) Capacitance- and ohmic resistance-corrected OER activity curves of IrO_2_, PrBaCo_2_O_5+*δ*_ (PBC), PrBa_0.5_Sr_0.5_Co_1.5_Fe_0.5_O_5+*δ*_ (PBSCF) powders and PBSCF nanofibers in 0.1 M KOH at 10 mV s^−1^ with a rotation rate of 1,600 r.p.m. These curves were averaged from three independent measurements. (**b**) Tafel plots obtained from the steady-state measurements. (**c**) Mass activities and BET surface area-normalized intrinsic activities of catalysts at *η*=0.37 V derived from (**a**); error bars represent s.d. from three independent measurements. (**d**) OER activity comparison in 0.1 M KOH. *iR*-corrected overpotential (*η*) at 10 mA cm^−2^_disk_, Tafel slope and catalyst mass loading of PBSCF-0, III and IrO_2_ in this work are compared with recently reported advanced perovskite catalysts with novel compositions^11^,^12^,^13^,^14^, nanostructures^11^,^15^ and atmosphere-treated surfaces^17^,^18^; all *η* derived from literatures are *iR*-corrected, mass loading of Ca_0.9_Yb_0.1_MnO_3−*δ*_ is missing^17^ and is assumed to be the same to the lowest number in the figure; BSCF is Ba_0.5_Sr_0.5_Co_0.8_Fe_0.2_O_3−*δ*_; *iR*-correction is very important for reliable comparison of results from different test devices (Supplementary Fig. 13; Supplementary Table 3; Supplementary Note 6). (**e**) Chronopotentiometric curves of the PBSCF-III nanofiber and commercial IrO_2_ catalysts at 10 mA cm^−2^_disk_. (**f**) High-resolution TEM images and the corresponding FFT patterns of the PBSCF-III after ink preparation process (ultrasonic treatment for 5 h), and chronopotentiometry test at 10 mA cm^−2^_disk_ for 1 h and 12 h. Scale bar, 5 nm.

To evaluate the OER activity, we loaded the catalysts on a glassy carbon (GC) electrode, which was mounted to a rotating disk electrode (RDE) fixture for measurements in 0.1 M KOH solution. All potentials were calibrated with respect to reversible hydrogen electrode (RHE; Supplementary Fig. 7a; Supplementary Note 2). Figure 2a shows typical OER activity curves of the as-synthesized catalysts, together with that of a commercial IrO_2_ (with a surface area of 146.68 m^2^ g^−1^) for direct comparison. The data were corrected for the effect of the ohmic resistance and capacitance (Supplementary Fig. 7b; Supplementary Note 3). Tafel plots shown in Fig. 2b were obtained from the steady-state measurements^30^, which are similar to the plots derived from capacitance-corrected cyclic voltammetry (CV) curves (Supplementary Fig. 8; Supplementary Note 4). The PBSCF-0 powder catalyst exhibited lower onset potential, higher kinetic current density at a fixed potential (Fig. 2a) and lower Tafel slope (67 mV dec^−1^; Fig. 2b) compared to those of PBC-0 powder, suggesting that the OER activity and kinetics are enhanced by the co-doping into PBC. Further, the PBSCF-0 powders exhibited higher mass activity (that is, catalyst mass loading-normalized kinetic current density) and ∼4.7 times intrinsic activity (that is, the BET surface area-normalized kinetic current density) at *η*=0.37 V than those of the PBC-0 powders (Fig. 2c), confirming the positive effect of the co-doping on intrinsic activity. Furthermore, the effect of the Co to Fe ratios in PrBa_0.5_Sr_0.5_Co_2-x_Fe_x_O_5+*δ*_ (*x*=0, 0.5, 1, 1.5 and 2) on the intrinsic activity was investigated. While the activities for the samples with *x*=0.5, 1 and 1.5 are similar, they are better than those for the samples with *x*=0 and 2 (Supplementary Fig. 9). Moreover, the PBSCF-0 has a much higher intrinsic activity than that of the commerical IrO_2_, as shown in Fig. 2c. However, the IrO_2_ has a lower onset potential, lower *η* of 0.394 V at 10 mA cm^−2^_disk_ (which is a metric related to solar fuel synthesis^31^; Fig. 2a), lower Tafel slope (59 mV dec^−1^; Fig. 2b) and much higher mass activity (Fig. 2c) than those of the PBSCF-0. The relation of higher intrinsic activity but a lower mass activity relative to IrO_2_ is usually observed for perovskite oxide catalysts since they had a low specific surface area due to a necessary high-temperature calcination process.

To improve the mass activity, which is more important than intrinsic activity for practical applications, we fabricated nanofibers of PBSCF and characterized their electrochemical performances under the same conditions, as shown in Fig. 2. Compared to the PBSCF-0 powders, the ∼196 nm PBSCF-I nanofibers had a significantly decreased onset potential, enhanced kinetic current densities at fixed potentials (Fig. 2a) and much lower Tafel slope (58 mV dec^−1^; Fig. 2b), suggesting faster reaction kinetics due to the decreased feature size and improved surface area. The electrochemical performances, especially mass activity (Fig. 2c), of the PBSCF-I nanofibers were already comparable to those of IrO_2_ catalyst. By further decreasing the diameter of the fibers to ∼83 and ∼20 nm, the electrochemical performance was further increased (Fig. 2a,b). The PBSCF-III nanofiber (∼20 nm in diameter) delivered a current density of 10 mA cm^−2^_disk_ at an *η* of 0.358 V (versus RHE), and a Tafel slope of 52 mV dec^−1^, which is much lower than those for IrO_2_ catalyst. The mass activity was markedly enhanced by increasing the specific surface area of the catalysts (Fig. 2c). The mass activity at *η*=0.37 V of the PBSCF-I, II and III nanofibers was ∼6.3, ∼9.5 and ∼20 times higher than that of the PBSCF-0 powder, respectively. For the PBSCF-III nanofiber, its mass activity was ∼72 times higher than that of the PBC-0 powder, and still ∼2.5 times higher than that of the commercial IrO_2_ catalyst (where the intrinsic activity is ∼20 times higher). The mass activity depended linearly on the surface area for PBSCF-0, I and II samples (Supplementary Fig. 10a), suggesting that they have similar intrinsic activity (Fig. 2c). However, the mass activity of the PBSCF-III nanofiber was greater than the value predicted from the surface area effect (Supplementary Fig. 10a), indicating that the intrinsic activity of PBSCF-III is greater than that of other three PBSCF samples (Fig. 2c). The intrinsic activity of the PBSCF-III was ∼1.6 times higher than that of the PBSCF-0 catalyst at *η*=0.37 V, suggesting that there are some intrinsic difference (for example, electronic structure) between the PBSCF-III nanofiber and the PBSCF-0 powder.

The OER performance of the ultrafine PBSCF-III nanofiber was superior to that of the recently reported advanced perovskite catalysts with novel compositions^11^,^12^,^13^,^14^, nanostructures^11^,^15^ and atmosphere-treated surfaces^17^,^18^ in terms of *iR*-corrected *η* (*i* is the current, *R* is the ohmic resistance), Tafel slope and catalyst mass loading in 0.1 M KOH (Fig. 2d, for example, *η*=0.358 V, Tafel slope of 52 mV dec^−1^, 0.202 mg_oxide_ cm^−2^_disk_ for PBSCF-III are lower than *η*=∼0.55 V, Tafel slope of 129 mV dec^−1^, 0.64 mg_oxide_ cm^−2^_disk_ for surface-modified BSCF (calcined at 950 °C under O_2_)^18^ and *η*=0.49 V, Tafel slope of 69 mV dec^−1^, 0.25 mg_oxide_ cm^−2^_disk_ for ∼80 nm LaCoO_3_ (ref. 15)), indicating that ultrafine PBSCF-III nanofiber is an outstanding electrocatalyst for OER. In addition, chronopotentiometry was used to evaluate the stability of the PBSCF-III nanofiber catalyst for 12 h (Fig. 2e). The potential from PBSCF-III almost remained unchanged throughout the test for initial 1 h, whereas the potential from IrO_2_ catalyst increased gradually. There was little observable change for PBSCF-III in the electrochemical impedance spectra and the cyclic voltammograms before and after chronopotentiometric test for 1 h (Supplementary Fig. 10b). Moreover, there were no significant changes (only a small, gradual increase) in the potential during the 12 h test for the PBSCF-III nanofiber catalyst, suggesting that the catalyst has high stability under the OER condition. Further evidence of the stability was from TEM analysis shown in Fig. 2f. It has been reported that the ink preparation conditions may significantly change the surface structure of perovskites such as BSCF^10^. Unlike BSCF^10^ but similar to the previously reported PBC^19^, little surface amorphization was observed for PBSCF-0 (Supplementary Fig. 11) and PBSCF-III (Fig. 2f) catalysts after ink preparation. No further change in the surface was observed after the stability test for 1 and 12 h (Fig. 2f). The continuous CV measurements demonstrate that all PBSCF catalysts have relatively high stability (Supplementary Fig. 12; Supplementary Note 5).

### Density functional theory calculations for OER

**Figure 3:**
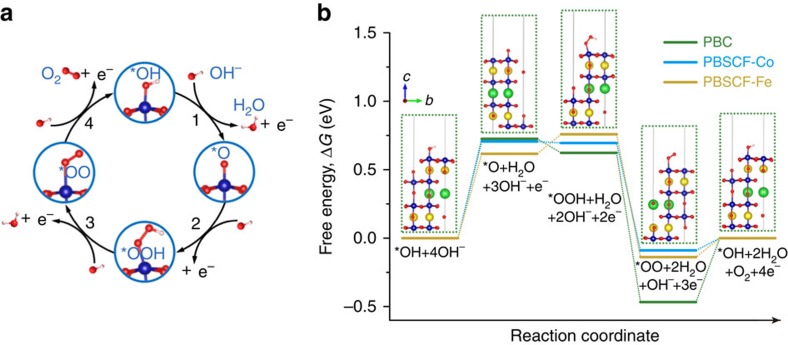
Density functional theory calculation for the OER mechanism. (**a**) Schematic of a four-step OER mechanism on perovskite oxide catalyst in the alkaline electrolyte. (**b**) The computed Gibbs free energy changes for the whole system for the OER at *η*=0 V/298.15 K on a PrBaCo_2_O_5+*δ*_ (PBC)/PrBa_0.5_Sr_0.5_Co_1.5_Fe_0.5_O_5+*δ*_ (PBSCF) surface; the B-site in PBC (Co) and PBSCF (Co/Fe) is treated as the catalytically active centres, all adsorbates are bonded to these redox centres; insets are atomic structures of PBC slabs with adsorbates. In all schematics, each deep blue ball is Co or Fe, fluorescent yellow ball is Pr, green ball is Ba or Sr, red ball is O and light pink ball is H.

To understand the atomic and electronic level mechanism of the effect of Sr, Fe co-doping in PBC on OER activity, we resorted to first-principles calculations of the surface OER reactions for PBC and PBSCF (the oxidation states of Pr are +3 (Supplementary Fig. 14; Supplementary Note 7), with more details in the Supplementary Methods and Supplementary Note 8). Since the B-sites (that is, the sites occupied by Co and Fe) are believed to be the catalytically active centres^1^, we examined the surfaces terminated with both CoO and FeO using (001) surface planes (Supplementary Fig. 15). Shown in Fig. 3a are the four-step, four-electron OER reactions based on the previously reported OER mechanism in an alkaline medium^8^, where the OER reaction started on catalyst adsorbed with *OH (see detailed reason in the Supplementary Information), unlike some other reported OER mechanisms (inverse ORR process) including a step with a bare catalyst surface^4^,^32^,^33^. The Gibbs free energy of reactions in the whole system at 298.15 K and 1 atm. pressure were estimated from density functional theory (DFT) calculations, via thermodynamic and electric-potential energy corrections with respect to the reversible hydrogen electrode; the methodology has been extensively used for a diverse range of catalytic processes^34^,^35^,^36^,^37^. Four different adsorbates *O, *OOH, *OO and *OH on Co or Fe were evaluated (Supplementary Fig. 16). At the equilibrium potential under standard conditions (that is, *η*=0 V at 298.15 K), the OER energy profiles for both PBC and PBSCF are shown in Fig. 3b. All three adsorption scenarios share very similar energy profiles, where the step 1 from *OH to *O and step 4 from *OO to *OH+O_2_ are uphill/endothermic, indicating that an external driving force (for example, electrical potential) is necessary to drive the OER reaction. Once the reaction reaches *OOH, it becomes downhill/exothermic and will automatically covert to *OO adsorbate. The difference in free energy of these steps and the similar energy profiles indicate that co-doping Sr and Fe can influence the OER kinetics, but the same reaction mechanism still remains. The step with the largest barrier is referred to as the potential-determining step, determining the overpotential^38^,^39^,^40^. For all three adsorption scenarios, the step 1 from *OH to *O is the potential-determining step. PBSCF with Fe as adsorption site shows the lowest overpotential requirement, that is, 0.6 V to start the reaction. For PBC and PBSCF Co site, the overpotential needs to exceed ∼0.7 V to make OER occur. The theoretical calculation clearly demonstrates that the Sr/Fe co-doping in PBC enhances the OER activity on the Fe site, consistent with the electrochemical measurement results.

It was reported that the OER stability is closely related to the position of the computed O *p*-band centre relative to the Fermi level^19^. To gain some insight into the stability of PBSCF, its O *p*-band centre was calculated and compared with those for the reported representative perovskites (Supplementary Fig. 17). Similar to the LnBaCo_2_O_5+*δ*_ (ref. 19), the O *p*-band centre of PBSCF is neither too close nor too far away from the Fermi level, implying high stability.

## Discussion

**Figure 4:**
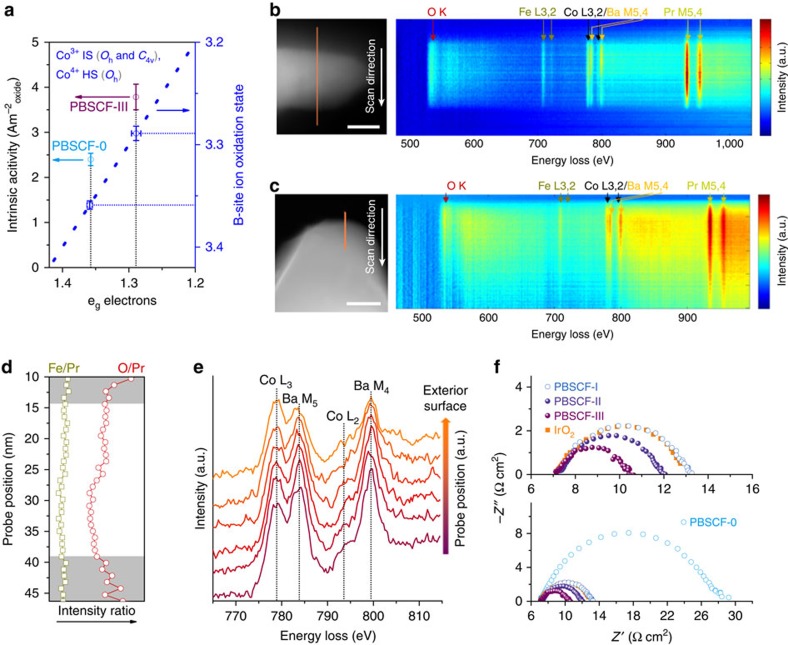
Structural homogeneity and charge transfer properties of PBSCF samples. (**a**) Intrinsic activity versus e_g_ electron filling of PBSCF-0, III; the bold blue dot line indicates the relationship between e_g_ electron filling and cobalt (B-site) oxidation state by a first-order approximation that the Co^3+^ is in intermediate spin (IS) in the octahedral (*O*_h_) and square pyramidal (*C*_4v_), and the Co^4+^ is in high spin (HS) in the *O*_h_ symmetry^19^. Error bars represent s.d. from three independent measurements. (**b**,**c**) STEM images including the electron energy-loss spectroscopy (EELS) scan lines (orange) across (**b**) the PBSCF-III nanofiber and (**c**) the PBSCF-0 particle, together with EELS line scan signal profiles in two-dimensional mode; scale bar (white line), 20 nm (**b**), 100 nm (**c**). (**d**) O/Pr and Fe/Pr intensity ratio in the fiber (the grey area indicates the surface region of nanofiber with obvious different O/Pr ratios), and (**e**) EELS spectra of Co L_2,3_ and Ba M_4,5_ ionization edges (acquired from surface region) of PBSCF-III. (**f**) Electrochemical impedance spectra of PBSCF-0, I, II, III and IrO_2_ catalysts recorded at 1.658 V (versus RHE) in 0.1 M KOH with a rotation rate of 1,600 r.p.m.

Increasing specific surface area by reducing particle size of catalysts is a well-known strategy to improve mass activity, as demonstrated for Ba_0.5_Sr_0.5_Co_0.8_Fe_0.2_O_3−*δ*_ (ref. 1) and SrNb_0.1_Co_0.7_Fe_0.2_O_3−*δ*_ (ref. 14). However, the intrinsic activity degraded as the surface areas were increased for these cases. In our study, the significantly enhanced mass activity of the ultrafine PBSCF-III nanofiber is attributed not only to the increased surface area but also to improved intrinsic activity. Very recently, Zhou *et al*.^15^ reported the nanosize effect of single perovskite cobaltite on their electronic structures and found the existence of surface spin-state transitions. The ∼80 nm LaCoO_3_ with the e_g_ electron filling of ∼1.2 exhibited the best performance. However, for double perovskites, the presence of octahedral (*O*_h_) and square pyramidal (*C*_4v_) symmetries and multiple spin configurations for B-site ions lead to the difficulty in identifying their spin states^19^. By a first-order approximation that the Co^2+^ (3d^7^), Co^3+^ (3d^6^) and Co^4+^ (3d^5^) in the double perovskite are in high spin (HS) in the *C*_4v_ symmetry, intermediate spin (IS) in the *O*_h_ and *C*_4v_, and HS in the *O*_h_ symmetry, respectively, Grimaud *et al*.^19^ proposed a relationship between e_g_ electron filling and cobalt (B-site) oxidation state obtained by chemical titration (Fig. 4a) and found higher OER activity for the double perovskites with the e_g_ electron filling close to ∼1.26, almost consistent with the previously reported descriptor of surface cation e_g_ electron filling^1^.

The B-site ion oxidation state of PBSCF-0, and III was determined by iodometric titration (Supplementary Table 4), and the corresponding e_g_ electron filling was estimated from the relationship proposed by Grimaud *et al*.^19^ (Supplementary Note 9). As the PBSCF-III has an e_g_ electron filling (∼1.29) closer to ∼1.26 compared to that of PBSCF-0 (∼1.36), PBSCF-III has higher intrinsic OER activity than PBSCF-0 (Fig. 4a). The B-site ion oxidation state from chemical titration represents the mean value in the entire perovskite. It was reported that the oxidation states of B-site ions on the surface may be different from that in the bulk^41^. Accordingly, scanning TEM (STEM) electron energy-loss spectroscopy line scan was acquired to characterize the variations in surface composition and charge distribution in PBSCF-III (Fig. 4b) and PBSCF-0 (Fig. 4c). Summarized in Fig. 4d are the O/Pr and Fe/Pr intensity ratios, which can be correlated with the oxygen vacancy concentration distribution^42^ and/or surface oxygen-containing adsorbates. Increased O/Pr ratio from inner to exterior in the surface layer of 5–10 nm width were observed in both PBSCF-III (Fig. 4d) and PBSCF-0 (Supplementary Fig. 18a), where the Fe/Pr ratio almost remained unchanged. If the O/Pr ratio variation is mainly contributed to oxygen vacancy concentration, an increased Co oxidation state from inner to exterior should be present, which means that the Co L_3_/L_2_ white line ratio decreases correspondingly^43^. However, both PBSCF-III (Fig. 4e) and PBSCF-0 (Supplementary Fig. 18b) demonstrate that the Co L_3_/L_2_ white line ratio increases from inner to exterior in the surface layer, suggesting that O/Pr ratio variation is due mainly to surface oxygen-containing adsorbates. It reveals that the PBSCF-III has more oxygen-containing adsorbates on the surface compared to that of PBSCF-0, suggesting its higher adsorption ability for oxygen-containing adsorbates, which may be beneficial to OER activity. Also, PBSCF-III has a somewhat higher Co L_3_/L_2_ white line ratio in the surface (that is, lower Co oxidation state) compared to that of PBSCF-0, which may make its e_g_ electron filling in the surface further close to the optimal value. However, it is difficult to quantify the Co L_3_/L_2_ ratio to understand the oxidation state due to the strong overlap with Ba M_4,5_. In addition, Fig. 4b indicates an inhomogeneous element distribution for PBSCF-III, in which the Pr M_4,5_ white line reached the highest intensity in the near middle probe position but Ba M_5,4_ white line intensity is low in the corresponding position, suggesting the presence of minor heterostructure in the PBSCF-III compared to PBSCF-0 with homogeneous element distribution (Fig. 4c). Such minor heterostructure may result in a synergistically enhanced OER activity^44^.

While the relationship between e_g_ electron filling and cobalt (B-site) oxidation state proposed by Grimaud *et al*.^19^ is used to explain the origin of enhanced OER activity of the PBSCF-III nanofiber, there still exists some uncertainty in spin state of Co ions in double perovskite. For example, it has been reported that Co^4+^ is likely in HS state in some double perovskites^45^,^46^ and La_1–*x*_Sr_*x*_CoO_3_ (ref. 47), but the Co^4+^ in SrCoO_3−*δ*_ was found to be in IS state^48^,^49^. If the Co^4+^ is in an IS state and Co^3+^ remains in an IS state in PBSCF, then the enhanced intrinsic activity of PBSCF-III over that of PBSCF-0 will be mainly attributed to other properties (rather than e_g_ electron filling) such as stronger adsorption of oxygen-containing adsorbates, surface reduction and heterostructure in PBSCF-III as revealed by electron energy-loss spectroscopy.

Moreover, compared to the PBSCF-0 powders, the PBSC-I, II and III nanofibers had markedly decreased charge transfer resistance (Fig. 4f). This is different from the reported case by decreasing the size from bulk to 200–60 nm nanoparticles, where the resistances of nanoparticles were relatively close to that of the bulk^15^, suggesting that the fast charge transfer of PBSCF nanofibers is attributed mainly to their unique nanofiber morphology. The ultrafine PBSCF-III nanofiber exhibited the highest rate of charge transfer, much better than that of the IrO_2_ catalyst. The combined features of optimized structure/surface electronic structure, high surface area and ultrafast charge transfer resulted in attractive intrinsic activity and markedly enhanced mass activity for ultrafine PBSCF-III nanofiber, indicating that it is a very promising candidate of the next-generation electrocatalysts for OER.

In summary, experimental measurements and DFT calculations reveal that the A/B site co-doped double perovskite PBSCF has a better OER activity than that of PBC due mainly to the enhanced activity of the Fe site. With increasing specific surface area by reducing particle size of PBSCF from bulk to nanofibers (∼196 and 83 nm in diameters), the OER mass activity increased while the intrinsic activity remained unchanged. However, the PBSCF-III nanofiber with a diameter of ∼20 nm demonstrated a mass activity ∼20 times and intrinsic activity ∼1.6 times higher than that of the PBSCF-0 catalyst at *η*=0.37 V, superior to that of IrO_2_. The markedly enhanced OER activity and stability of the ultrafine PBSCF-III nanofiber could be attributed to the unique nanostructure, favourable surface electronic structure, high surface area, high surface oxygen species and possible presence of some heterostructures. This work not only results in a highly efficient and durable electrocatalyst for OER, which may have important technological implications, but also offers new insight into the development of advanced materials by nanostructure engineering for other applications of energy storage and conversion.

## Methods

### Materials synthesis

All chemicals were used as received without any further purification procedure.

The PrBa_0.5_Sr_0.5_Co_1.5_Fe_0.5_O_5+*δ*_ (PBSCF) nanofibers were prepared via electrospinning method followed by calcination process. In a typical procedure, taking PBSCF-II as an example (details for other samples see Supplementary Table 1), stoichiometric amount of Pr(NO_3_)_3_·6H_2_O (1 mmol, Alfa Aesar, 99.9%), Ba(NO_3_)_2_ (0.5 mmol, Alfa Aesar, ACS, 99+%), Sr(NO_3_)_2_ (0.5 mmol, Alfa Aesar, 99.97%), Co(NO_3_)_2_·6H_2_O (1.5 mmol, Alfa Aesar, ACS, 98−102%) and Fe(NO_3_)_3_·9H_2_O (0.5 mmol, Alfa Aesar, ACS, 98−101%) were dissolved in *N*,*N*-dimethylformamide (DMF, 7.5 ml) under vigorous stirring in an ∼80 °C oil bath. Ba(NO_3_)_2_ was ground by mortar and pestle before adding into DMF to make it easier to dissolve. Then, PVP (Fluka Analytical, Sigma–Aldrich, K90, Mw 360,000; 15 wt.% based on the mass of DMF) powders were added into the above solution, which was further stirred overnight at room temperature to ensure the PVP was fully dissolved. The as-obtained precursor solution was loaded into a plastic syringe equipped with a 25-G needle for electrospinning. The applied voltage and distance between the needle tip and the collector was fixed at 18 kV and 15 cm, respectively. The feeding rate for the precursor solution was controlled by programmable syringe pump (New Era Pump Systems Inc.) and was fixed at 2 μl min^−1^. An aluminum foil was wrapped on a rotating metal drum as a collector. The relative humidity in the electrospinning chamber was controlled to be 30–40%. The as-obtained electrospun fibers were calcined under air at 700−750 °C for 3 h with heating and cooling rates of 1 and 3 °C min^−1^, respectively.

PBSCF and PBC powders were synthesized using a Pechini process. Stoichiometric amounts of Pr(NO_3_)_3_·6H_2_O, Ba(NO_3_)_2_, Sr(NO_3_)_2_, Co(NO_3_)_2_·6H_2_O and Fe(NO_3_)_3_·9H_2_O were dissolved in deionized water with proper amount of citric acid. An adequate amount of ethylene glycol was added into the solution after the mixture was dissolved. After a viscous resin was formed, the mixture was heated to roughly 250 °C in air and followed by combustion to form fine powders, which were calcined at 600 °C for 4 h and then 1,000 °C for 4 h. The resulting powders were then ground. The PBC powders were further calcined at 1,100 °C for 2 h to form a pure phase. Finally, the powders were ground and sieved through a 170-mesh sieve before using.

### Basic characterization

The phase structures of samples were characterized by X-ray diffraction with an X'Pert PRO Alpha-1 X-ray diffractometer. The microstructures, morphologies and structures of samples were analysed by a scanning electron microscope (LEO 1530) and a high-resolution TEM (FEI G2 Tecnai F30). High-angle annular dark-field scanning transmission electron micrographs (STEM) and chemical investigations were carried out using the same TEM equipped with a Gatan GIF system (Tridiem 863 UHS). Before TEM analysis, the samples were ultrasonically dispersed in ethanol. The BET-specific surface areas and corresponding Barrett–Joyner–Halenda pore size distribution plots were derived from the nitrogen adsorption–desorption isotherm measurements at the boiling point of liquid nitrogen (77 K) using a Micromeritics ASAP 2020 analyser. X-ray photoelectron spectroscopy (XPS) measurement was performed on a Thermo K-Alpha XPS spectrometer (Thermo Fisher Scientific) equipped with a monochromatic Al-Kα X-ray source (*hv*=1468.6 eV). Oxygen content and the mean oxidation state of the B-site cations in the perovskite oxides were evaluated by iodometry method (with details in the Supplementary Methods).

### Electrochemical measurement

Working electrodes were prepared by drop-casting catalyst ink on a GC (5 mm in diameter, 0.196 cm^2^ in geometric area) RDE (Pine Instrument Company, USA). GC insert was polished with 0.05 μm alumina and rinsed by ethanol before catalyst drop-casting. The catalyst ink was a mixture of 4.0 mg of catalyst, 1.0 mg of acetylene black carbon (sieved through a 170-mesh sieve before using) and 20.0 μl of Nafion solution (5 wt.% D-521, Alfa Aesar) dispersed in 2.00 ml of 3:1 (v/v) deionized water/isopropanol mixed solvent. To be homogeneous, the catalyst ink was ultrasonically treated for 5 h or more. To form a catalyst thin film, 20.0 μl of catalyst ink was transferred onto the surface of GC in RDE, dried in an oven at 70 °C for ∼5 min and completely dried under fume hood at room temperature, yielding a catalyst mass loading of 0.202 mg_oxide_ cm^−2^_disk_. The same mass loading was used for all catalysts including electrospun nanofibers, perovskite powders and commercial IrO_2_ for all electrochemical measurements.

Electrochemical measurement was performed on a Solartron electrochemical workstation (Solartron SI 1287 electrochemical interface and SI 1255 HF frequency response analyser) with a RDE system (Pine Instrument Company, USA) using a 150 ml glass cell at room temperature. A Ag/AgCl electrode prefilled with 4 M KCl aqueous solution saturated with AgCl was used as the reference, and the as-measured potentials (versus Ag/AgCl) were calibrated with respect to RHE (Supplementary Fig. 7a; Supplementary Note 2). A Pt wire electrode was used as the counter electrode. A 0.1 M KOH aqueous solution diluted from 1.0 M KOH standard solution (Fluka Analytical, Sigma–Aldrich) was used as an electrolyte. Oxygen (ultra-high purity grade, Airgas) was bubbled into the electrolyte to make it O_2_-saturated during the measurements to ensure the O_2_/H_2_O equilibrium at 1.23 V versus RHE. Before the electrochemical measurement, the drop-casted catalyst on GC-RDE was well wetted carefully using a dropper with 0.1 M KOH solution. CV tests were carried out at 10 mV s^−1^ with a rotation rate of 1,600 r.p.m. The CV curve was then capacitance- and ohmic resistance-corrected to get the OER activity curve (Supplementary Fig. 7b; Supplementary Note 3). Potential stair-step measurement was performed with potentiostatic steps of 20 mV every 30 s, which is referred to as steady-state measurements^30^. Electrochemical impedance spectroscopy measurements were performed in the frequency range of 100 kHz–50 mHz at 0.7 V versus Ag/AgCl with a rotation rate of 1,600 r.p.m., and the a.c. modulation was controlled at 10 mV. Chronopotentiometry tests were performed at a constant current density of 10 mA cm^−2^_disk_ with a rotation rate of 1,600 r.p.m. Before all electrochemical measurement, the catalyst was electrochemically activated via CV test between 0.2 and 1.0 V (versus Ag/AgCl) at a scan rate of 100 mV s^−1^ for ∼15 cycles to obtain reproducible curves (Supplementary Fig. 12; Supplementary Note 5).

### Data availability

The data that support the findings of this study are available from the corresponding author on request.

## Additional information

**How to cite this article**: Zhao, B. *et al*. A tailored double perovskite nanofiber catalyst enables ultrafast oxygen evolution. *Nat. Commun.* **8**, 14586 doi: 10.1038/ncomms14586 (2017).

**Publisher's note**: Springer Nature remains neutral with regard to jurisdictional claims in published maps and institutional affiliations.

## Supplementary Material

Supplementary Information Supplementary Figures, Supplementary Tables, Supplementary Notes, Supplementary Methods and Supplementary References
