# Supplementary material for: A tailored double perovskite nanofiber catalyst enables ultrafast oxygen evolution
Source: Nat Commun. 2017 Feb 27;8:14586. doi: 10.1038/ncomms14586 (PMC5333368; doi:10.1038/ncomms14586)
Supplement: Supplementary Information — Supplementary Figures, Supplementary Tables, Supplementary Notes, Supplementary Methods and Supplementary References [file ncomms14586-s1.pdf]

Supplementary Information

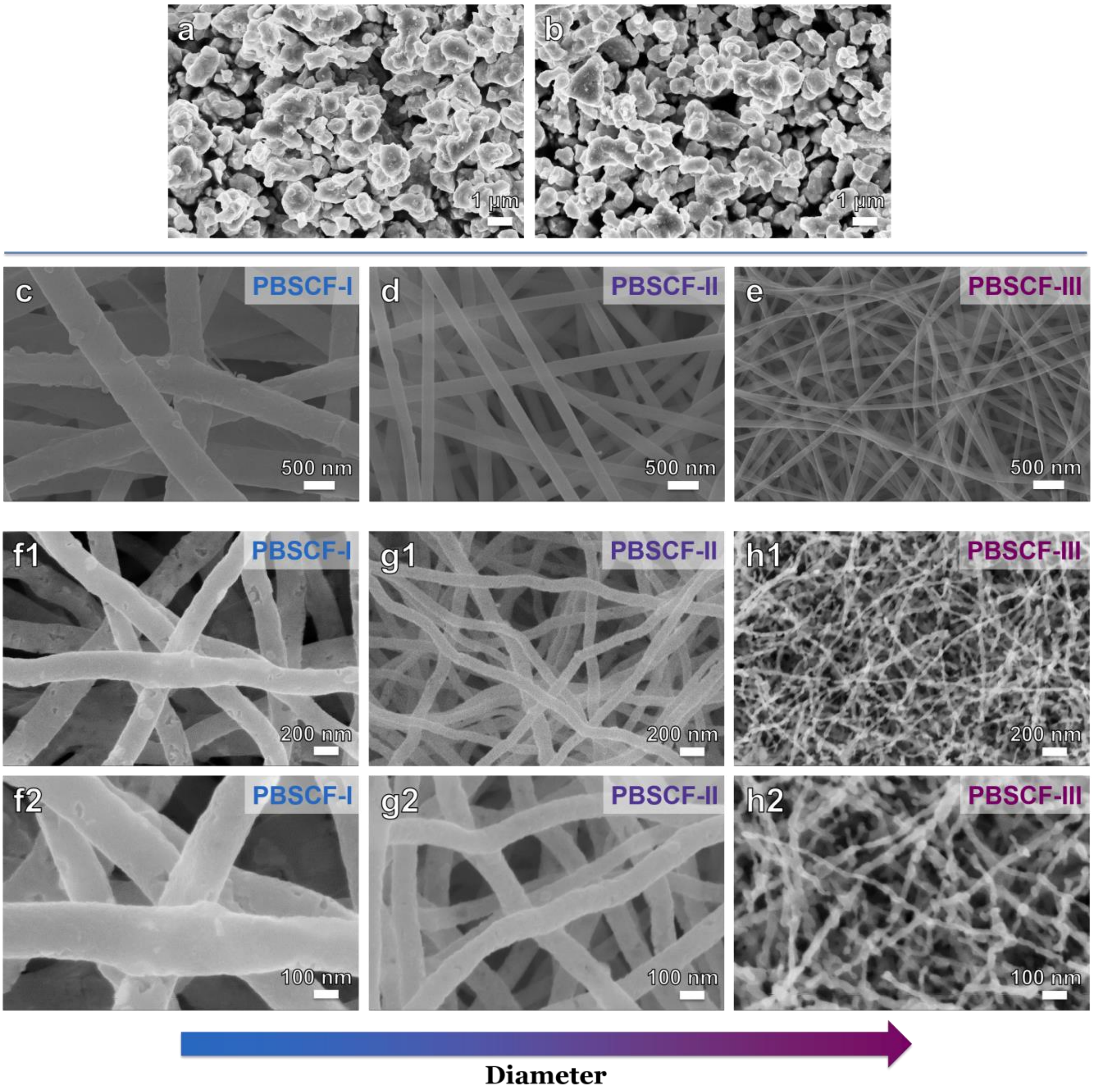

Supplementary Figure 1. SEM images of samples. (a) PBC-0, and (b) PBSCF-0 powders after calcination; (c-e) electrospun PBSCF precursor fibers with controlled diameters before calcination: (c) PBSCF-I, (d) PBSCF-II and (e) PBSCF-III; (f-h) PBSCF nanofibers with controlled diameters after calcination at 750 ℃ for 3 h: (f1, f2) PBSCF-I, (g1, g2) PBSCF-II and (h1, h2) PBSCF-III. After calcination, all fibers shrank with diameter decreased compared to precursor fibers.

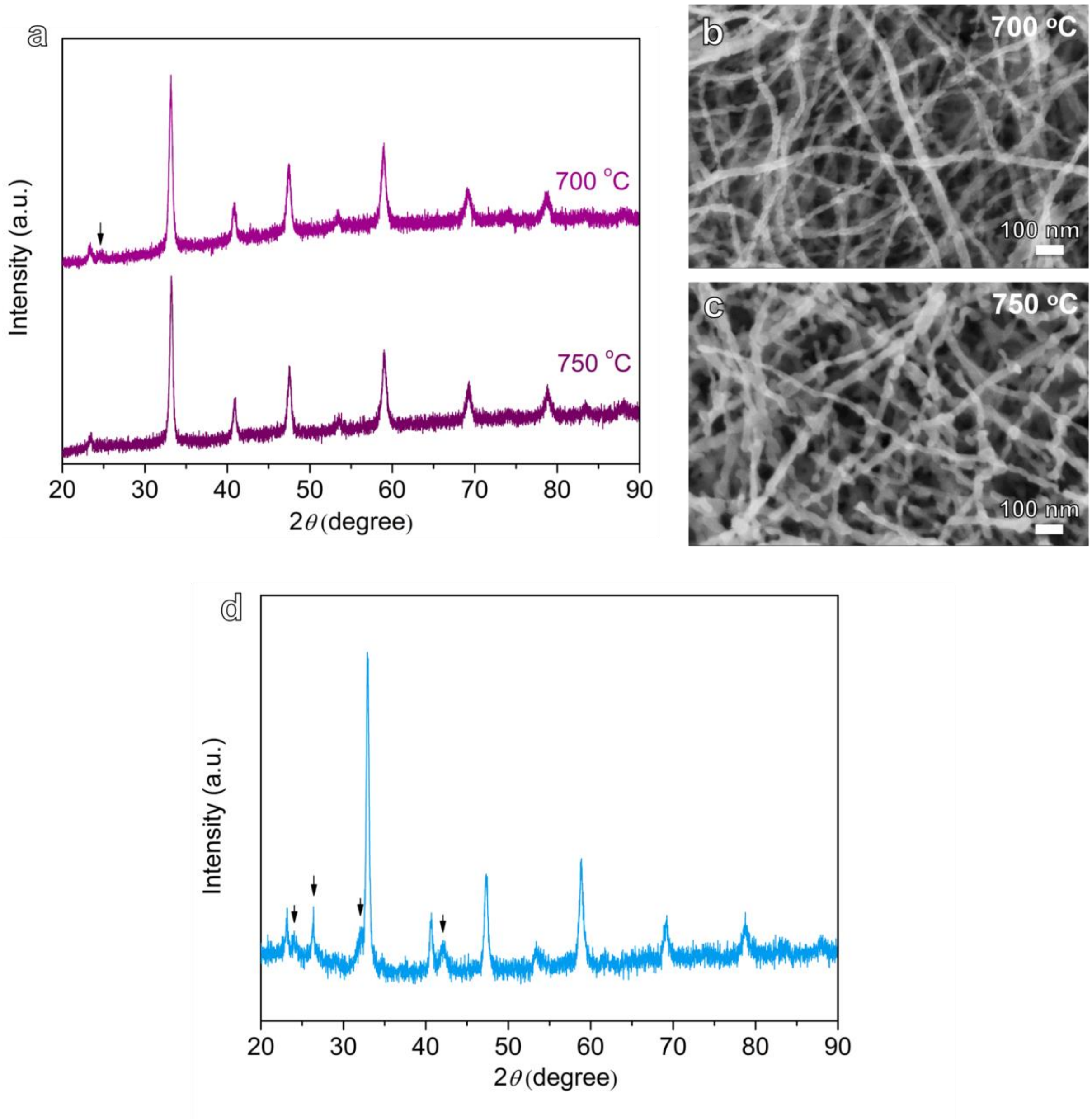

Supplementary Figure 2. (a) XRD patterns and (b, c) SEM images of PBSCF-III nanofibers calcined at 700 and 750 ℃ for 3 h; (d) XRD pattern of PBSCF powders prepared using a Pechini process after calcination at 600 ℃ for 4 h and then 750 ℃ for 3 h; the arrows indicate the presence of impurities.

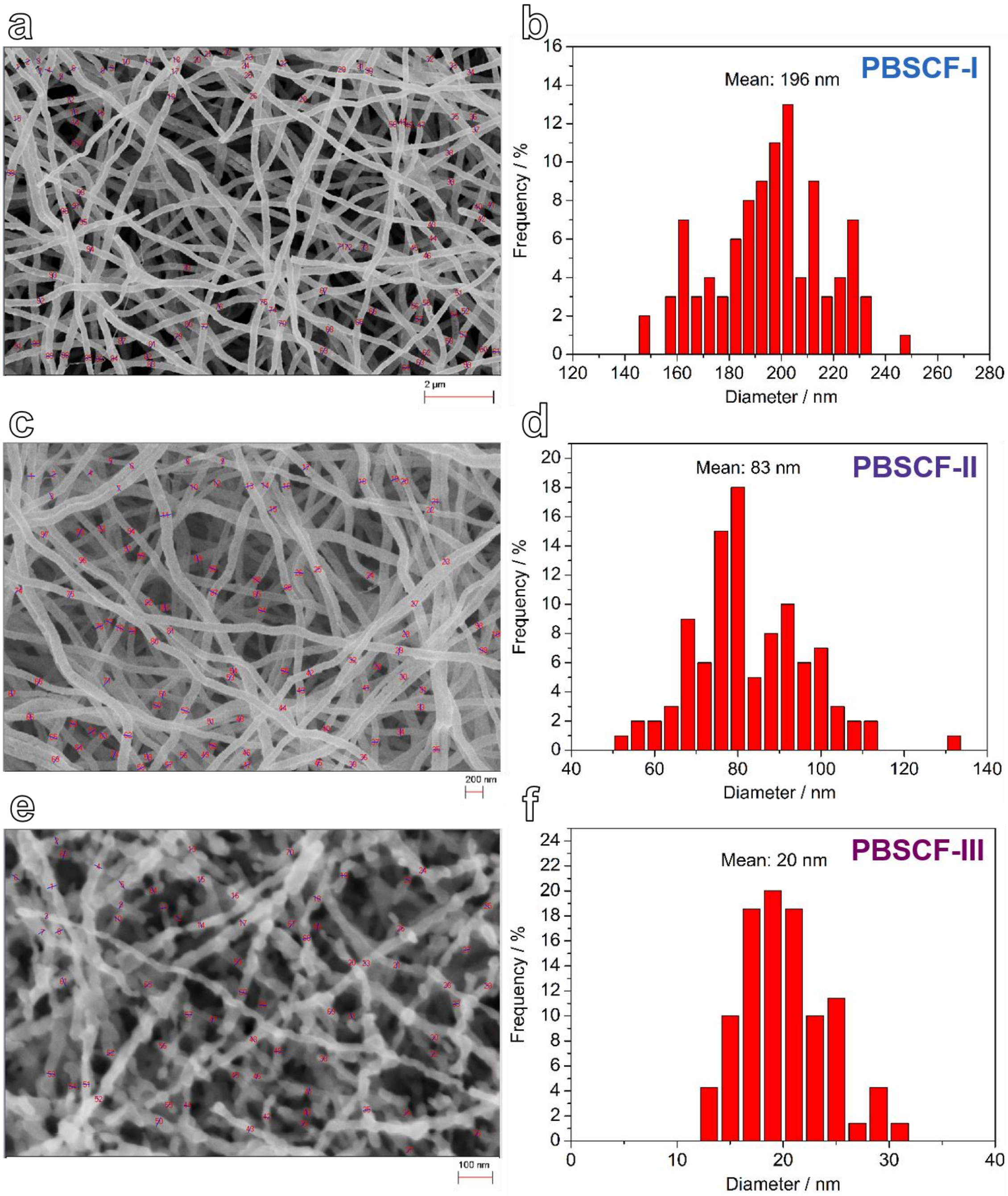

Supplementary Figure 3. (a,c,e) SEM images and (b,d,f) the corresponding diameter distribution of PBSCF nanofibers with controlled diameters after calcination at 750 ℃ for 3 h: (a, b) PBSCF-I, (c, d) PBSCF-II and (e, f) PBSCF-III.

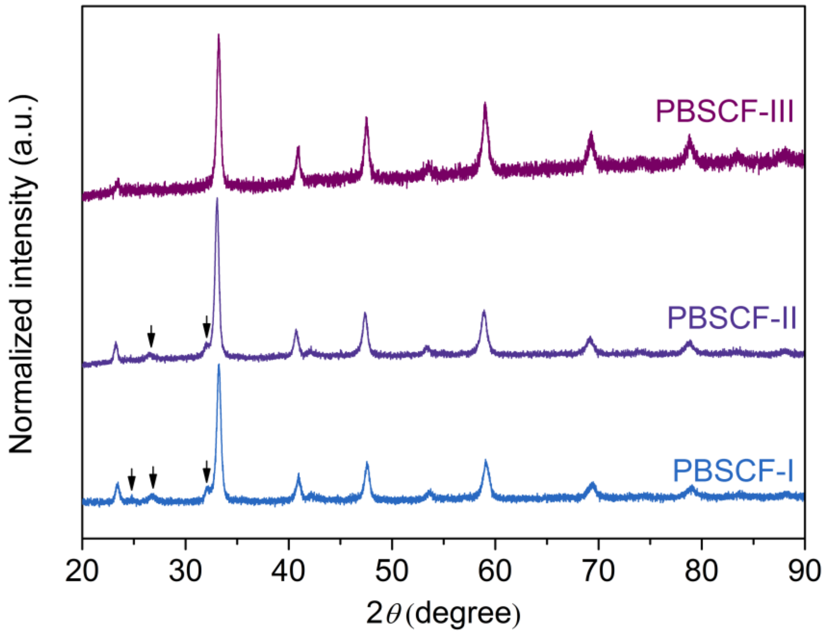

Supplementary Figure 4. XRD patterns of PBSCF-I, PBSCF-II, and PBSCF-III nanofibers calcined at 750 ℃ for 3 h; the arrows indicate the presence of minor impurity.

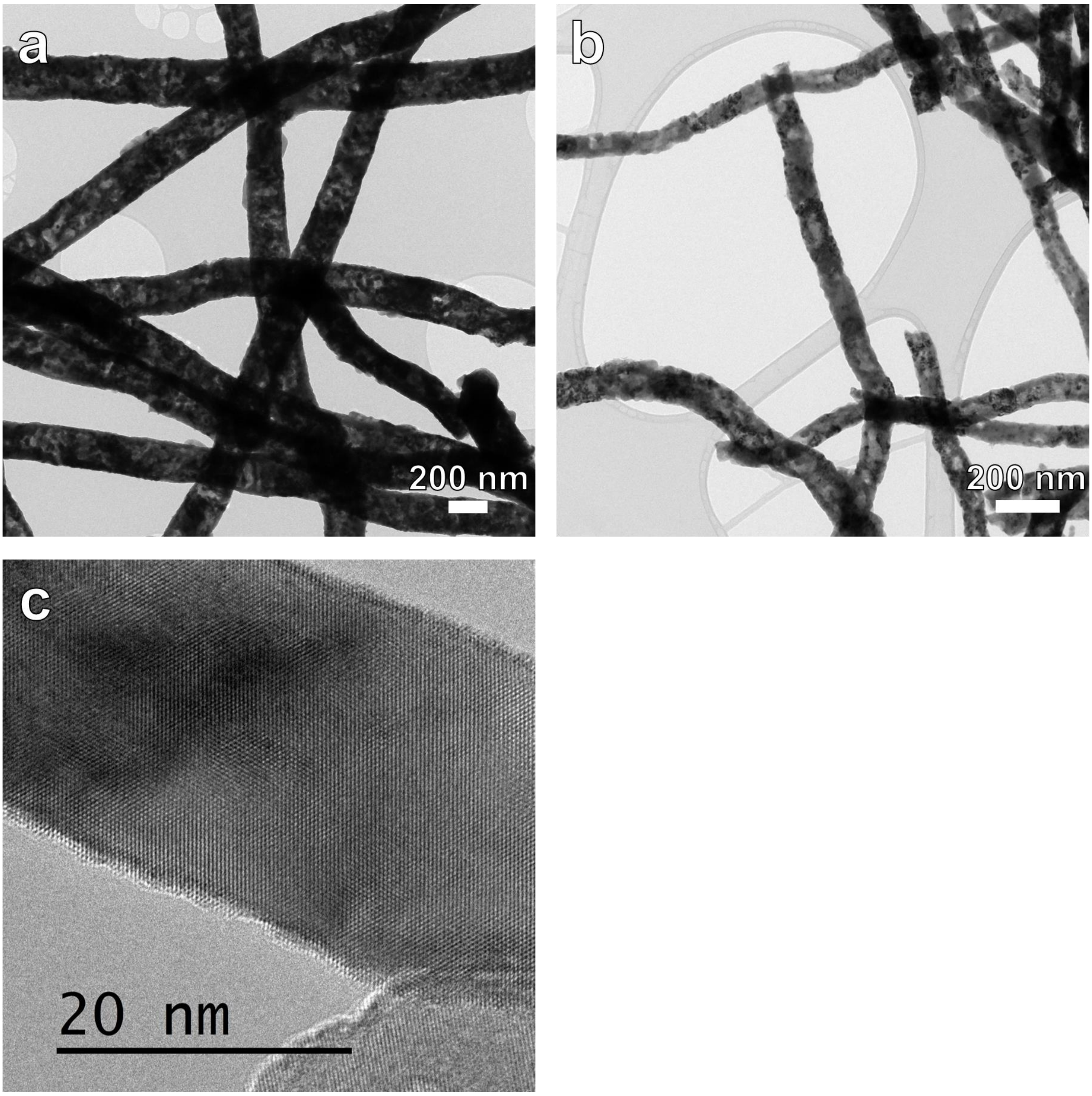

Supplementary Figure 5. (a, b) TEM images of (a) PBSCF-I and (b) PBSCF-II nanofibers after calcination at 750 ℃ for 3 h; (c) HRTEM image of PBSCF-III nanofiber after calcination at 750 ℃ for 3 h.

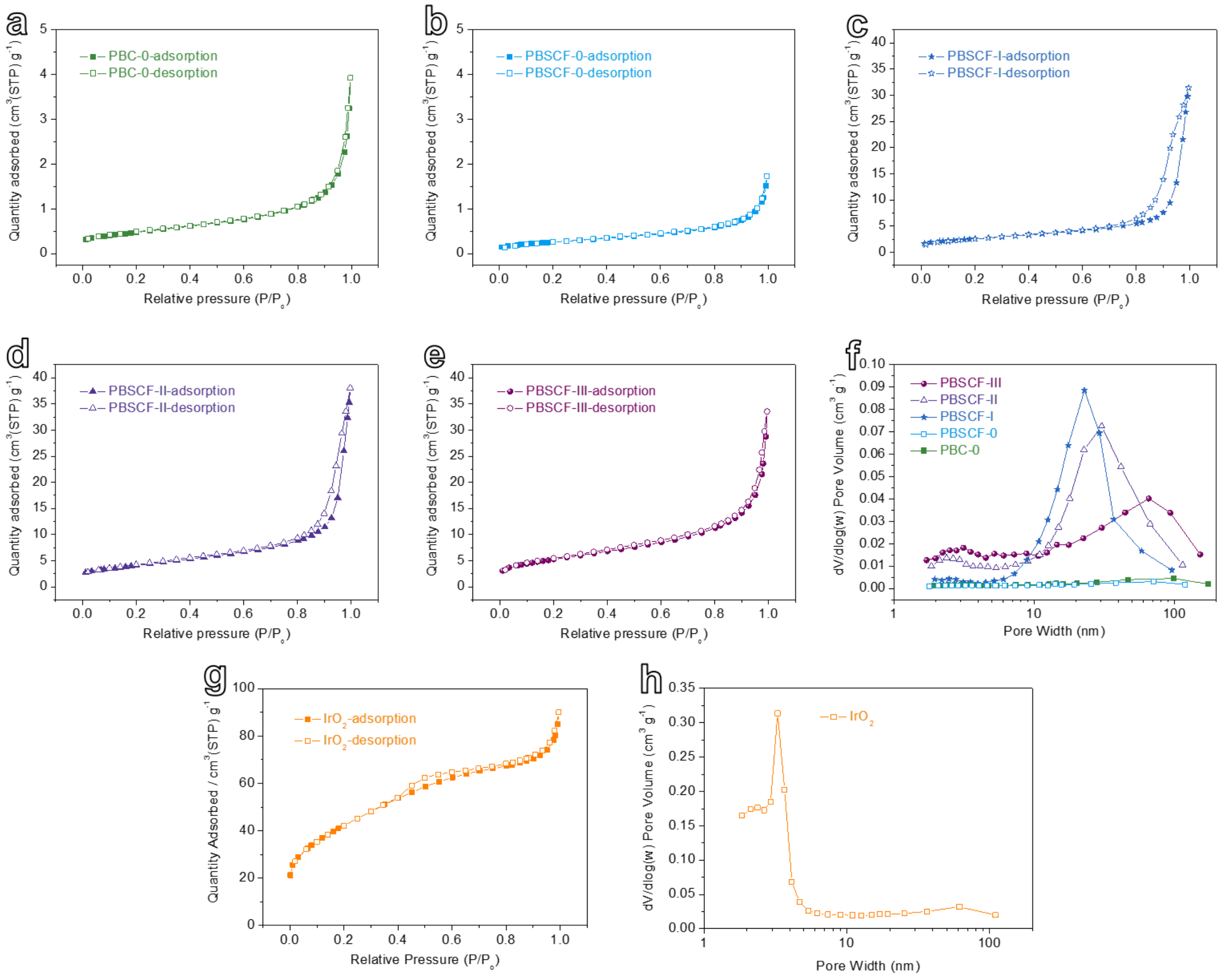

Supplementary Figure 6. (a-e) The nitrogen adsorption–desorption isotherms, and (f) corresponding Barrett–Joyner–Halenda (BJH) pore size distribution plots of as-calcined samples: (a) PBC-0, (b) PBSCF-0, (c) PBSCF-I, (d) PBSCF-II, and (e) PBSCF-III; (g) The nitrogen adsorption–desorption isotherms, and (h) corresponding BJH pore size distribution plot of commercial IrO_2.

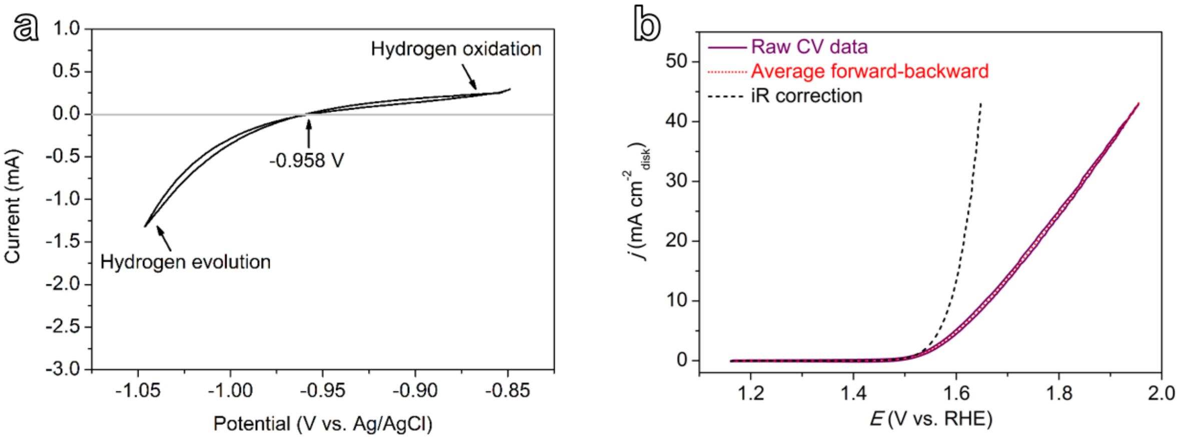

Supplementary Figure 7. (a) CV curve of Pt mesh working electrode at a scan rate of 1 mV s^{-1} in the ultra high purity H_2-saturated 0.1 M KOH solution for RHE calibration of the Ag/AgCl reference electrode; (b) Capacitive and ohmic corrections of the as-measured CV curve (10 mV s^{-1}) of example catalyst (i.e., PBSCF-III).

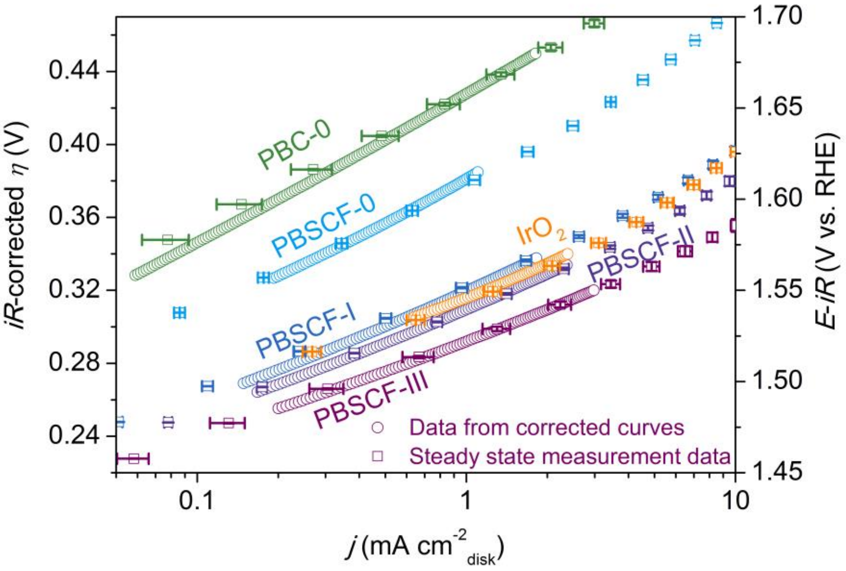

Supplementary Figure 8. Comparison between Tafel plots (hollow circle) derived from the capacitance- and ohmic resistance-corrected OER activity curves as shown in **Figure 2a** and Tafel plots obtained from the steady-state measurements (hollow square). Error bars represent s. d. from three independent measurements.

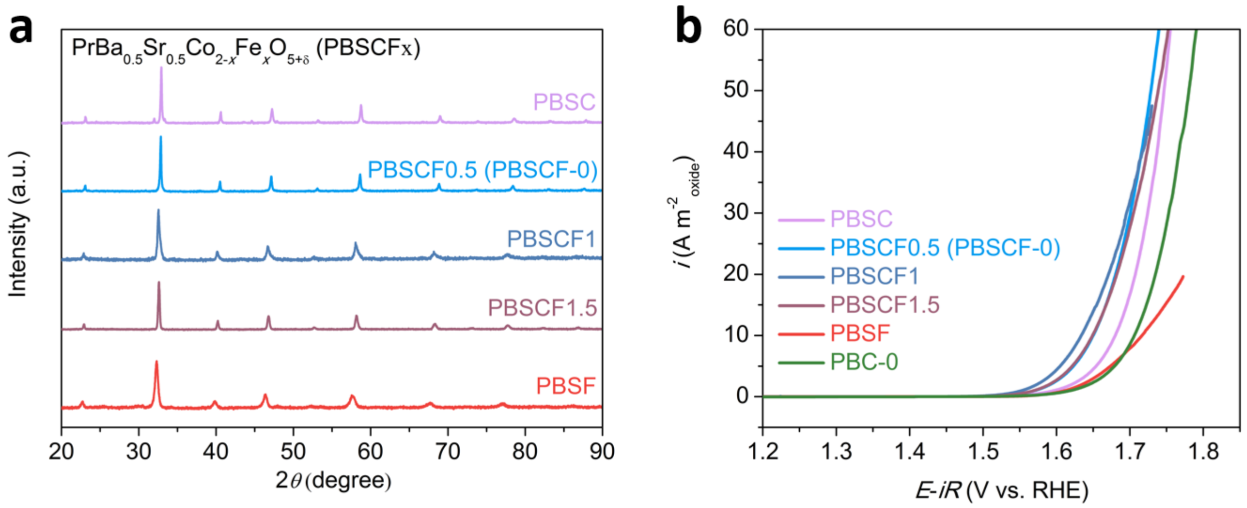

Supplementary Figure 9. (a) XRD patterns, and (b) capacitance- and ohmic resistance-corrected OER intrinsic activity (normalized by the BET surface area, **Supplementary Table 2**) curves in 0.1 M KOH at 10 mV s^{-1} with a rotation rate of 1,600 rpm of $PrBa_{0.5}Sr_{0.5}Co_{2-x}Fe_xO_{5+\delta}$ (x = 0, 0.5, 1, 1.5, and 2, named PBSCF*x*, that is, PBSC, PBSCF0.5, PBSCF1, PBSCF1.5, and PBSF, respectively) powders synthesized by the Pechini process. The powders were finally calcined at 1000 or 1100 ℃ to obtain relatively pure phase.

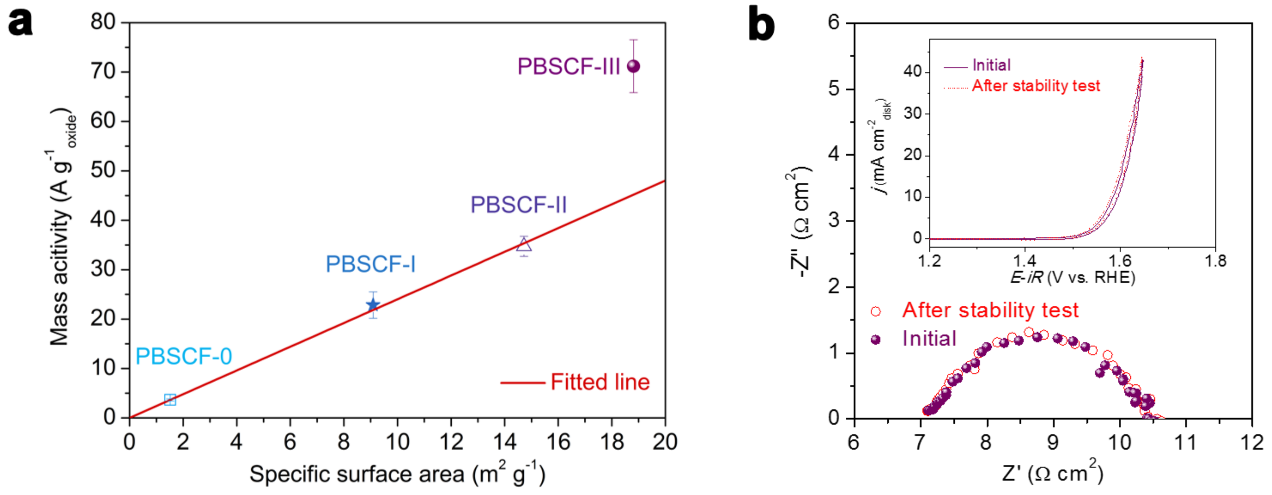

Supplementary Figure 10. (a) The relationship between mass activity at η = 0.37 V and specific surface area of PBSCF samples. Error bars represent s. d. from three independent measurements. (b) Electrochemical impedance spectra of PBSCF-III catalyst recorded at 1.658 V (vs. RHE) before and after chronopotentiometry test for 1 h; the inset shows the corresponding cyclic voltammograms at 10 mV s^{-1}.

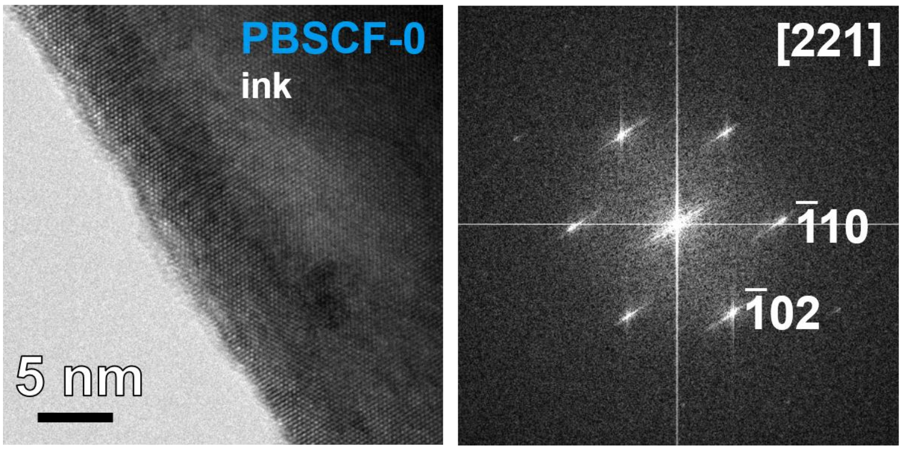

Supplementary Figure 11. HRTEM image and corresponding FFT pattern of PBSCF-0 after ink preparation process (ultrasonic treatment for 5 h).

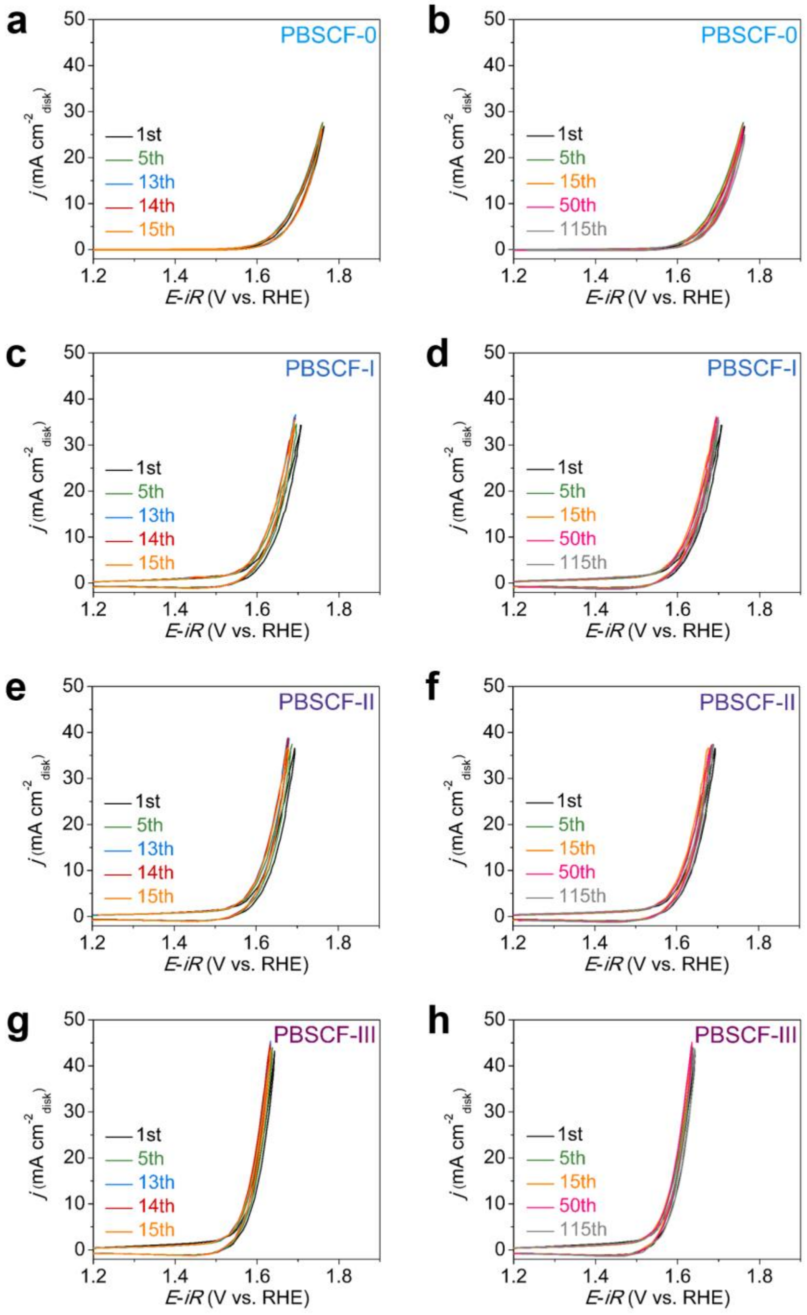

Supplementary Figure 12. CV curves of catalyts in 0.1 M KOH at 100 mV s^{-1} with a rotation rate of 1,600 rpm: (a,b) PBSCF-0, (c,d) PBSCF-I, (e,f) PBSCF-II and (g,h) PBSCF-III.

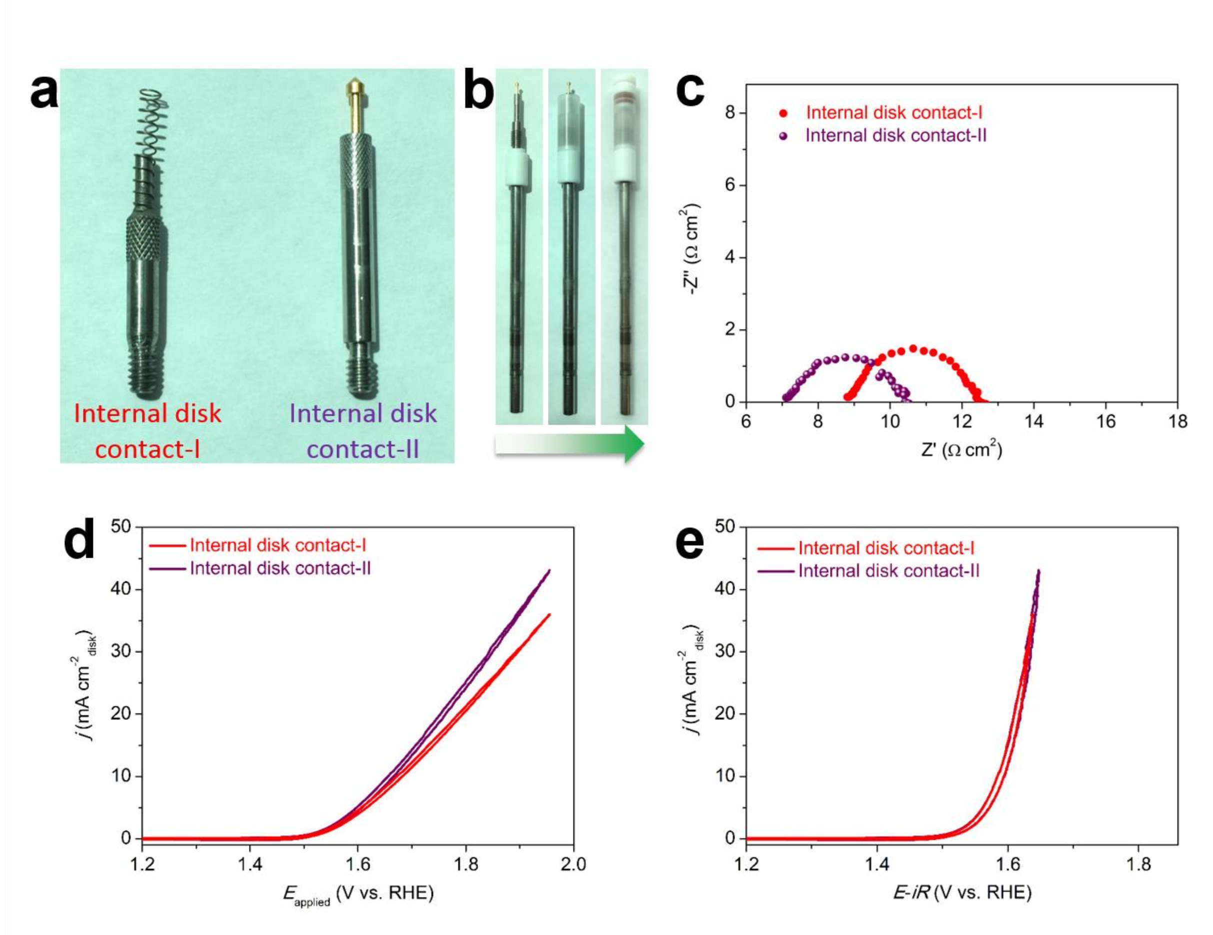

Supplementary Figure 13. Comparative study on the effect of different internal disk contact studs on the ohmic resistance and OER activity: (a) digital photos of two different internal disk contact studs (see details in **Supplementary Table 3**); (b) digital photos of assembly process for connecting the internal disk contact stud and RDE Tip onto a shaft; (c, d, e) electrochemical performance of PBSCF-III catalyst in an O_2-saturated 0.1 M KOH solution by using different internal disk contact studs: (c) electrochemical impedance spectra recorded at 1.658 V (vs. RHE), (d) CV curves at 10 mV s^{-1}, and (e) ohmic resistance-corrected OER activity curve from (d).

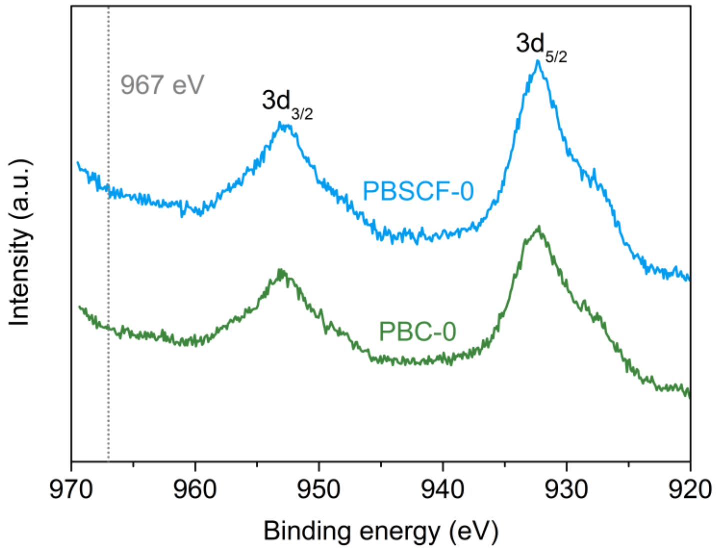

Supplementary Figure 14. XPS spectra of Pr 3d in PBC-0 and PBSCF-0.

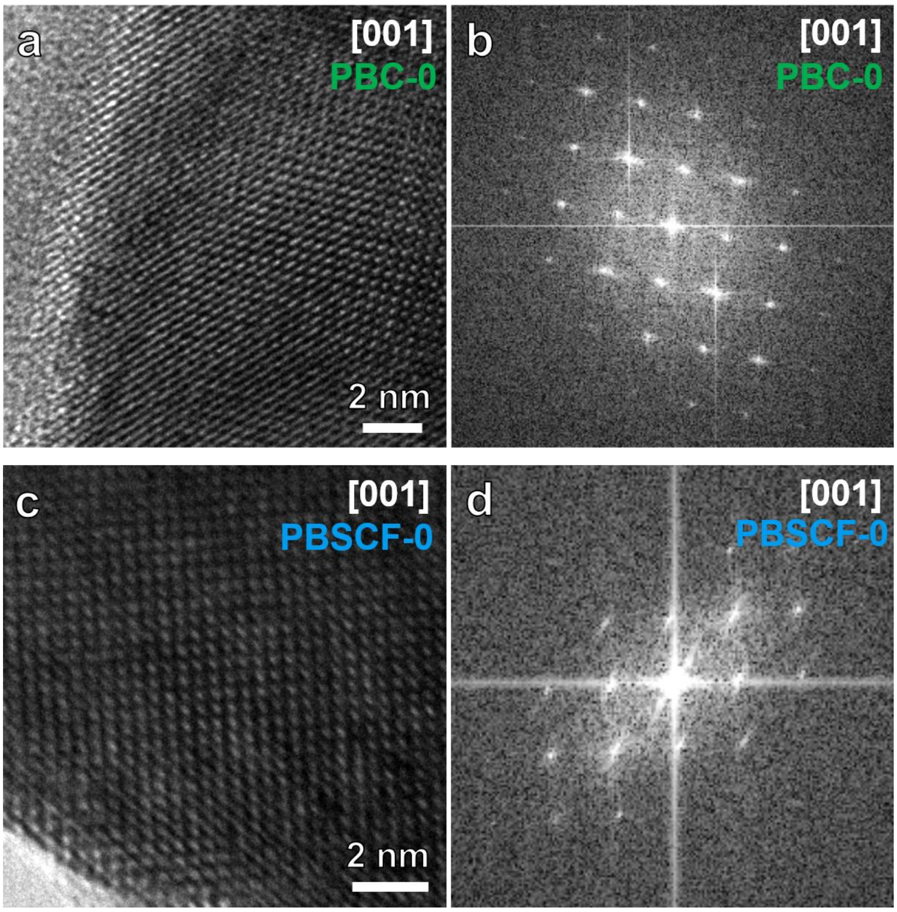

Supplementary Figure 15. (a, c) HRTEM images and (b, d) corresponding FFT patterns of (a, b) PBC-0 and (c, d) PBSCF-0.

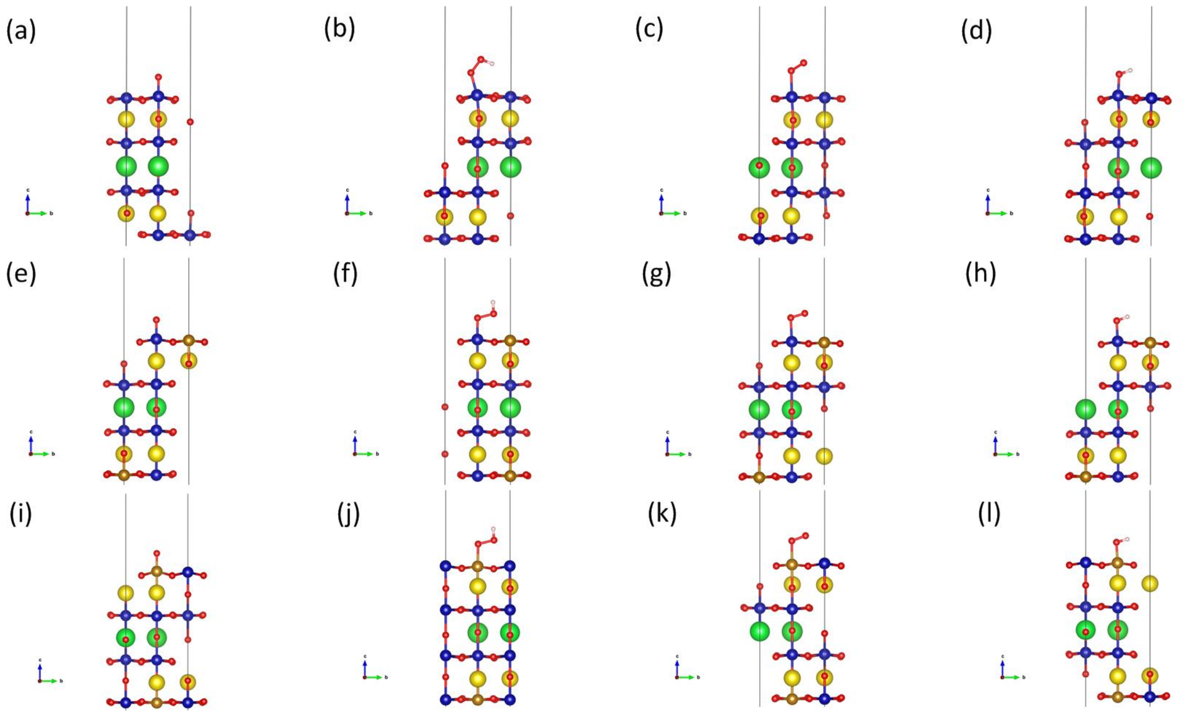

Supplementary Figure 16. Atomic structures of PBC and PBSCF slabs with adsorbed *O, *OOH, *OO, *OH. (Deep blue ball is Co, bronze ball is Fe, fluorescent yellow ball is Pr, green ball is Ba and Sr, red ball is O, and light pink ball is H) (a)~(d) is PBC with *O, *OOH, *OO, *OH; (e)~(h) is PBSCF with *O, *OOH, *OO, *OH on Co-site; (i)~(l) is PBSCF with *O, *OOH, *OO, *OH on Fe-site.

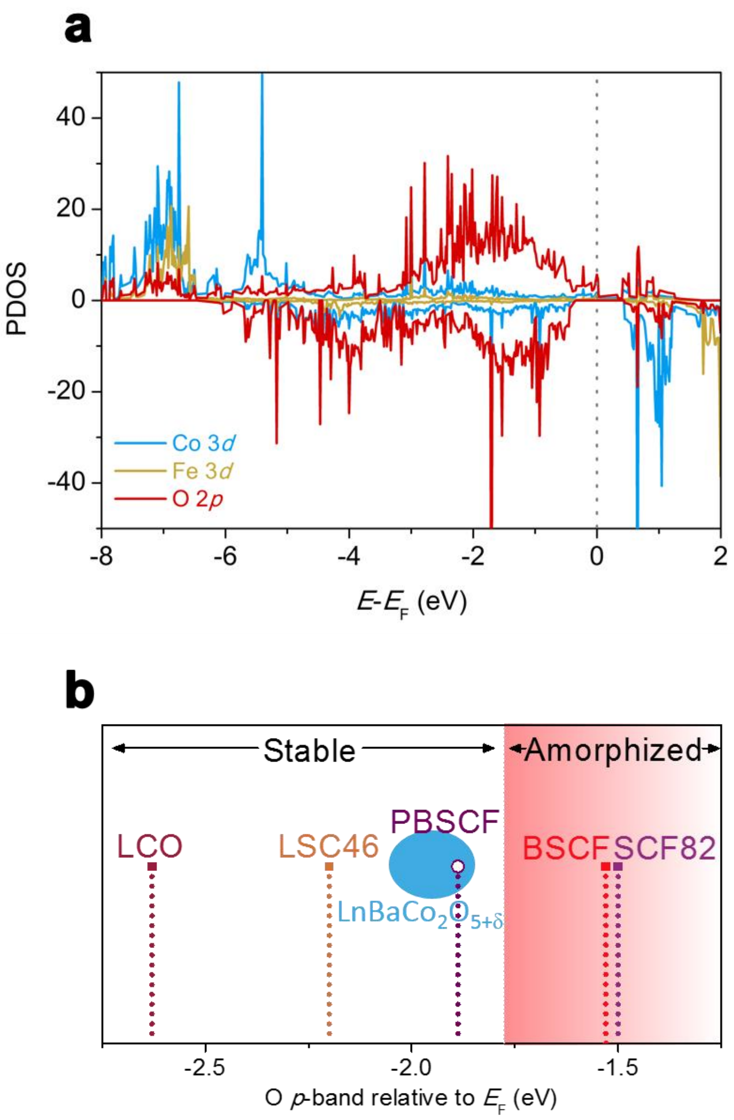

Supplementary Figure 17. (a) The partial density of states (PDOS) diagrams of the overall O p-band and Co, Fe d-band of PBSCF. (b) Structure stability versus the O *p*-band centre relative to E_F (eV) of PBSCF, reported $LnBaCo_2O_{5+\delta}$ (Ln = Pr, Sm, Gd and Ho), $LaCoO_3$ (LCO), $La_{0.4}Sr_{0.6}CoO_{3-\delta}$ (LSC46), BSCF, and $SrCo_{0.8}Fe_{0.2}O_{3-\delta}$ (SCF82)[1]; The relationship between stability and O *p*-band center was proposed in the literature[1].

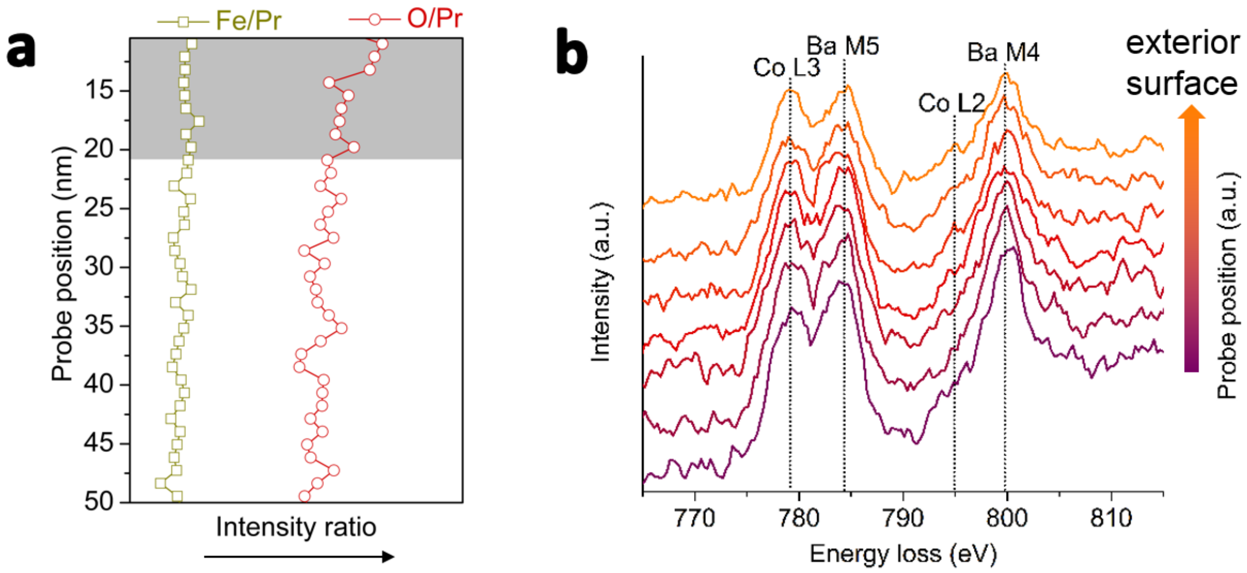

Supplementary Figure 18. (a) O/Pr and Fe/Pr intensity ratio in the particle (the grey area indicates the surface region with obvious different O/Pr ratios), and (b) EELS spectra of Co $L_{2,3}$ and Ba $M_{4,5}$ ionization edges (acquired from the surface region) of PBSCF-0.

Supplementary Table 1. Detailed electrospinning processing parameters for preparing PBSCF nanofibers.

Name	*n* /mmol	Solvent, DMF / mL	Polymer	Needle	Voltage /kV	Feeding rate /uL min^{-1}	Distance /cm	RH /%
PBSCF-I	1.6	7.5	18 wt.%PVP/DMF	21-G	18	5	15	30~40
PBSCF-II	1	7.5	15 wt.%PVP/DMF	25-G	18	2	15	30~40
PBSCF-III	0.1	7.5	15 wt.%PVP/DMF	25-G	18	2	15	30~40

Supplementary Table 2. BET specific surface areas of the catalysts.

Samples	Specific surface area / $m^2\ g^{-1}$
PBC-0	1.70
PBSC	1.32
PBSCF0.5 (PBSCF-0)	1.52
PBSCF1	3.30
PBSCF1.5	1.55
PBSF	6.51
PBSCF-I	9.09
PBSCF-II	14.72
PBSCF-III	18.81
Commercial IrO_2	146.68

$PrBa_{0.5}Sr_{0.5}Co_{2-x}Fe_xO_{5+\delta}$ (x = 0, 0.5, 1, 1.5, 2, named PBSCFx)

Supplementary Table 3. Detailed information of two different internal disk contact studs.

	Description	Supplier	Part#
Internal disk contact-I	Contact stud (without gold-plated)	Pine Instrument Company	ACE4TVQ
Internal disk contact-II	E4 replacement internal disk contact/contact stud probe (with gold-plated, spring-loaded pin contact)	Pine Instrument Company	ACE4TVP

Supplementary Table 4. B-site ion oxidation state and oxygen content (5+δ) in PBSCF-0, III samples.

Samples	B-site ion oxidation state	Oxygen content (5+δ)
PBSCF-0	3.359 ± 0.004	5.859 ± 0.004
PBSCF-III	3.289 ± 0.007	5.789 ± 0.007

Error is based on the standard deviation of triplicate measurements.

Supplementary Note 1. Phase purity of PBSCF prepared by different methods

As shown in **Supplementary Figure 2**, compared to the XRD pattern of PBSCF-III nanofiber calcined at 750 °Cfor 3 h, obvious impurity phase appeared in the XRD pattern of PBSCF bulk powders prepared using a Pechini process after calcination at 750 ℃ for 3 h (even with a pre-calcination process under 600 ℃ for 4 h), suggesting that the smaller particle size results in decreased calcination temperature to obtain a pure phase.

Supplementary Note 2. RHE calibration

All electrochemical measurements in this work were performed with a Ag/AgCl electrode as the reference electrode. The measured potentials were calibrated with respect to RHE (**Supplementary Figure 7a**). Briefly, a Pt mesh electrode was used as the working electrode in the system. CV curve was recorded at a scan rate of 1 mV s^{-1} in the ultra high purity H_2-saturated 0.1 M KOH solution. The average of the two potentials at current of 0 mA was taken to be the thermodynamic potential for the hydrogen electrode reactions. E (RHE) = E (Ag/AgCl) + 0.958 V.

Supplementary Note 3. Capacitive and ohmic corrections

To obtain a capacitance- and ohmic resistance-corrected OER activity curve, the as-measured CV curve (10 mV s^{-1}) is first capacitance-corrected by taking an average of forward and backward scan curves (**Supplementary Figure 7b**). Then the potentials of capacitance-corrected OER activity curve are ohmic resistance-corrected by subtracting iR (i is current, R is the ohmic resistance from EIS measurement) to obtain the final OER activity curve.

To get a reliable OER activity curve, three capacitance-corrected OER activity curves from independent experiments are taken an average. Then the average capacitance-corrected OER activity curve is further ohmic resistance-corrected.

Supplementary Note 4. Comparison of Tafel plots obtained from different methods

As shown in **Supplementary Figure 8**, Tafel plots (hollow circle) derived from the capacitance- and ohmic resistance-corrected OER activity curves are similar to the Tafel plots obtained from the steady-state measurements (hollow square); similar overpotentials were obtained at the same current density for both methods. The use of steady-state measurement (e.g., chronoamperometry or chronopotentiometry) mainly removes the capacitive contribution to current. In the Tafel plots derived from the corrected CV curves, the capacitive contribution is also properly removed (**Supplementary Figure 7b**). Thus, similar overpotentials are observed at the same

current density for both methods.

Supplementary Note 5. CV measurements of PBSCF catalysts

Prior to all electrochemical measurement, the catalyst was electrochemically activated via CV test for 15 cycles to obtain reproducible curves (**Supplementary Figures 12a,c,e,g**). All the catalysts were further run for another 100 cycles at 100 mV s^{-1} after the electrochemical activation process, with the results shown in **Supplementary Figures 12b,d,f,h**. The overlapped CV curves indicate that all the catalysts are relatively stable.

Supplementary Note 6. Importance of ohmic correction to performance comparison

The effect of different internal disk contact studs (with details given in **Supplementary Table 3**) on the ohmic resistance and OER activity was studied to understand the importance of iR-correction. As shown in **Supplementary Figure 13a**, the tip part of internal disk contact-II that will contact glassy carbon is gold coated, thus, it has a lower contact resistance. **Supplementary Figure 13c** shows the typical electrochemical impedance spectra by using different disk contact studs but the same materials under the same conditions. The high-frequency intercept (left-end) of the single impedance arc on the horizontal axis represents the total ohmic resistance. In some literatures, this is stated as ionic resistance[2] or electrolyte resistance[3], but it should be ohmic resistance including ionic/electrolyte resistance and contact resistance. The charge transfer resistances from the electrodes using internal disk contact-I and -II are almost the same but their ohmic resistances are obviously different, suggesting the difference of contact resistances as the electrolyte is the same. Accordingly, the CV curves appeared to be different (**Supplementary Figure 13d**). With ohmic resistance-correction, the OER activity curves overlapped, indicating the importance of iR-correction for understanding the activity contributed from the catalyst itself and for OER activity comparison with the results from literatures.

Supplementary Note 7. XPS analysis of Pr in PBC-0 and PBSCF-0

The oxidation states of Pr in the PBC-0 and PBSCF-0 samples were analyzed by XPS. The $3d_{5/2}$ peaks centered at 932.3 eV indicate that the Pr in the PBC-0 and PBSCF-0 samples should be +3 state and the absence of peaks or shoulds at 935.0~935.5 eV (characteristic $3d_{5/2}$ peak for Pr^{4+}) excludes the presence of Pr^{4+} in the samples[4]. Moreover, it's reported that the peak centered at ~967 eV can be used for qualitative assessment of the presence of Pr^{4+} in the samples[5, 6]. The absence of the peak at ~967 eV further confirms that the Pr in the PBC-0 and PBSCF-0 samples should be +3 state.

Supplementary Note 8. VASP POSCAR files for PBC and PBSCF slabs

```
PBC-slab
1.0
        5.4972262383         0.0000000000         0.0000000000
       -0.0276054081         5.4654854759         0.0000000000
        0.0109038002         0.0904106216        30.6688349064
   Ba    Pr    Co     O
    2     4     8    22
Cartesian
     2.755633048      0.043779785     11.860374363
    -0.008463301      2.777236089     11.859607515
     2.752178283      0.032659517      7.843462228
     2.755189326      0.057107136     15.904765984
    -0.010614332      2.767109833      7.844229076
     5.486397863      2.792540270     15.908966740
     0.003601235      0.032665728      5.967480769
     5.501689090      0.049541038     13.822106166
     2.740306458      2.764784426      5.998640028
     2.739394749      2.781433521     13.885928219
     0.005746557      0.039101672      9.813689684
     5.494172595      0.056021902     17.733610049
     2.740857962      2.772807034      9.882571655
     2.732136787      2.789541067     17.687392598
     0.006464405      0.044917393     11.964464625
     2.740238069      2.775195211     11.699393844
     4.198614000      4.218740372      6.050623921
     4.029145412      4.071233729     13.923068711
     1.279922042      1.302979617      6.050378511
     1.456524089      1.496503357     13.923037635
     4.013471927      1.496577007      6.046329937
     4.127501830      1.406601976     13.978762540
     1.461604989      4.024739613      6.045808956
     1.350859820      4.159558217     13.973978650
     4.180788673      4.205230808      9.731159828
     4.055193998      4.110136241     17.606762953
     1.298802132      1.333864842      9.728614331
     1.430896410      1.480602671     17.610044222
     0.004623786      0.029372817      7.881736997
     0.013212786      0.064795544     15.657667235
     2.739985073      2.764811742      7.980552549
     2.743804347      2.797796993     15.773197152
     4.118888136      1.405321293      9.658382378
     4.158232876      1.401694915     17.522056857
     1.360486219      4.133351981      9.656236300
     1.335982874      4.197840797     17.519541522
```

PBSCF-slab
1.0

5.5523290634	0.0000000000	0.0000000000
0.0039295995	5.5419316665	0.0000000000
0.0033827568	0.0016651100	25.6089436617

Ba	Sr	Pr	Co	Fe	O
1	1	4	6	2	22

Cartesian

0.005750992	2.772326449	11.720394192
2.779846002	0.001438609	11.726950132
0.004384367	2.771197939	15.757387567
5.552861214	2.771047700	7.736487137
2.777014523	0.001559817	15.721919858
2.780053114	5.541893321	7.762275867
5.556483102	5.542174129	5.944015050
0.003072775	0.001063864	13.719709986
2.780985087	2.771974050	13.631462004
5.557888746	5.542330576	17.555647427
0.001580415	0.000879579	9.778416538
2.780188212	2.772485680	9.739286203
2.779407748	2.771469237	17.458308134
2.777769711	2.772321302	5.930954687
5.553674411	0.001361874	11.569583161
2.782950984	2.774659951	11.813174855
4.258404292	1.295620131	5.960942577
4.246761294	1.280724778	13.737405681
1.299989563	4.246959133	5.959943922
1.316114233	4.264090660	13.736176919
1.437701362	1.439682614	6.045451995
1.418912920	1.369299044	13.786395873
4.120315155	4.103372964	6.042839923
4.144891587	4.173550457	13.795000258
4.132505487	1.418855198	17.367883488
4.156330478	1.376988193	9.562302549
1.426386267	4.123816614	17.369522854
1.404904939	4.168880335	9.563275637
5.557213953	5.540905454	7.911678020
0.006593150	0.004095448	15.597102097
2.782557613	2.773240187	7.789139192
2.776507803	2.769456273	15.480069037
4.138231929	4.119207246	17.389625700
4.066615793	4.093039091	9.650013235
1.420893974	1.424094069	17.394568222
1.493276564	1.452763035	9.648195278

Supplementary Note 9. Estimation of e_g electron filling in PBSCF samples

Grimaud et al.[1] proposed a relationship between e_g electron filling and cobalt (B-site) ion oxidation state (obtained by chemical titration) in double perovskites by a first order approximation that Co^{2+} is in high spin (HS) in the C_{4v} symmetry, Co^{3+} is in intermediate spin (IS) in the O_h and C_{4v}, and Co^{4+} is in HS in the O_h symmetry, respectively. The e_g electron filling of PBSCF samples in this work was estimated from the reported relationship between e_g electron filling and cobalt ion oxidation state using mean B-site ion oxidation state in PBSCF (**Supplementary Table 4**) as its Co:Fe ratio is relatively high (i.e., 3:1).

Supplementary Methods

Iodometry method

Oxygen content and the mean oxidation state of the B-site cations in the perovskite oxides were evaluated by iodometry method. Briefly, 1.0 g of KI and 20.00~21.00 mg of perovskite oxides were dissolved in a N_2-saturated HCl aqueous solution (4 mol L^{-1}) in sequence, under N_2 (ultra-high purity grade, Airgas) atmosphere in order to prevent the oxidation of iodide ions by air, and then the solution was titrated by 0.01 mol L^{-1} of $Na_2S_2O_3$ aqueous solution diluted from standard solution (0.05 mol L^{-1}, Fluka Analytical, Sigma-Aldrich). Towards the end point in the titration, 0.5 mL of starch solutions were added as an indicator. Pure N_2 was also bubbled during the titration process to obtain reliable results.

Computational Section

First-principles calculation was carried out using VASP (Vienna *Ab-initio* Simulation Package)[7]. The exchange-correlation functional used is the one adopting generalized gradient approximation parameterized by Perdew, Burke and Ernzerhof[8]. The core-electrons are treated in the projector augmented wave-function[9]. Valence electron configuration for each element is that $5s^25p^66s^2$ in Ba, $4s^24p^65s^2$ in Sr, $5s^25p^66s^24f^1$ in Pr (the other 2 f electrons are frozen in the core to mimic Pr^{3+} ion, the evidence of Pr oxidation states in the samples see **Supplementary Figure 14, Note 7**), $3d^84s^1$ in Co, $3d^74s^1$ in Fe, $2s^22p^4$ in O. Due to the strong-correlation of d electrons in Co and Fe, the Hubbard U correction[10] was applied to both elements with specific value 4 eV, according to the recent (La, Sr)(Co, Fe)$O_{3-\delta}$ calculations[11]. The spin polarization was applied on Co and Fe with spin structure as ferromagnetic for simplification. The $3 \times 3 \times 1$ Monkhorst-Pack[12] reciprocal grid was used to sample out the total energy numerically. Gaussian smearing with smearing width 0.01 was used to accelerate computation of electronic energy near Fermi level. Atomic positions were fully relaxed for both slab and the adsorbed radical using quasi-Newton algorithm[13], with electronic relaxation method the conjugate gradient algorithm for all band simultaneous update of orbitals[14]. The wave function kinetic energy cutoff was 520 eV for energy accuracy.

To avoid asymmetric slab induced dipole moments, all slabs were constructed symmetrically by adding one more layer of Co/Fe-O. The terminating layer is Co/Fe-O as Co/Fe was recognized as the catalytic active centers. All adsorbates, *O, *OH, *OO, and *OOH were bonded to these

redox centers. In order to calculate the energy of ions in solvent, the computational hydrogen electrode (CHE) [15] developed by Norskov et.al. was used as the reference electrode. Thus, proton and electron energy can be converted to the half energy of hydrogen molecule with an electric potential energy correction, i.e. –eU with U representing the electric potential with respect to the reversible hydrogen electrode (RHE). In this way, all electrochemical reaction energies can be mapped to the value with reference to RHE. In addition to 0 K electronic energy of every slab + adsorbate configuration, the 0 K to 298.15 K thermodynamic contribution was also added on. At 0 K, the zero-point vibrations of relevant gas molecules, i.e. H_2, O_2, H_2O, as well as the adsorbates *O, *OH, *OO, and *OOH were calculated by summing over the contributing vibration modes at Gamma point. Additionally, the 0 K to 298.15 K entropy and enthalpy parts for those molecules were also taken into consideration using NIST JANAF table[16] and the 0 K to 298.15 K free energies for those adsorbates were calculated via computed vibration modes in the vibrational partition function[15]. The externally applied electric field U can be varied to tune the reaction energies. The equation to calculate Gibbs reaction energy is shown below:

$$\Delta G_r = \Delta E_{0K} + \Delta ZPE + \Delta H_{0K\rightarrow 298.15K} - T\Delta S_{0K\rightarrow 298.15K} \pm neU \quad (1)$$

in which ΔE_{0K} is the reaction energy at 0 K, ΔZPE is the reaction energy from zero-point vibration, $\Delta H_{0K\rightarrow 298.15K}$ and $\Delta S_{0K\rightarrow 298.15K}$ are enthalpy and entropy contribution from 0 K to 298.15 K for reaction energy, $\pm neU$ is the electrochemical potential energy of n electrons (system reduction +, oxidation -) under electrode potential U. Since the PBE functional predicts over-binded triplet oxygen dimer, its energy at 298.15 K was calculated as $G_{O_2} = 2G_{H_2O} - 2G_{H_2} + 4 \times 1.23\ (eV)$.

The OER reaction mechanism is a 4-electron exchange mechanism:

$$*OH + OH^- \rightarrow *O + H_2O + e^- \quad (2)$$

$$*O + OH^- \rightarrow *OOH + e^- \quad (3)$$

$$*OOH + OH^- \rightarrow *OO + H_2O + e^- \quad (4)$$

$$*OO + OH^- \rightarrow *OH + O_2 + e^- \quad (5)$$

Note that the mechanism does not involve a step with a bare oxide catalyst surface. It was reported that metal oxides can adsorb species (e.g. H*, OH*, O*, etc.) from solution depending on the surface's pH_{pzc}[17, 18]. In the case with an electrolyte pH (~13) higher than the pH_{pzc} of oxides (~7–11 for binary oxides and perovskites), negatively charged adsorbate (OH^-) is expected to

accumulate on the catalyst surface[19].

For PBC and PBSCF, a 7-atomic layer slab with (001) plane Co/Fe-O exposed layer was constructed with over 15 Å vacuum layer, according to our experimental HR-TEM observation of (001) surface planes (**Supplementary Figure 15**). Although we do not know if the (001) plane is the dominating surface of PBC and PBSCF, the (001) plane was readily observed during our TEM analysis, suggesting that the (001) plane is one of the stable planes exposed on the surface. Additionally, the (001) surface with BO-transition metal ions (e.g., Co and Fe) has been widely used as the catalytically active and stable surface for simple perovskite in DFT calculations[20, 21]. We believe this is also the case for the double-perovskite catalysts PBC and PBSCF. Accordingly, a 7-atomic layer slab with (001) plane containing Co/Fe-O exposed to a vacuum layer (~15 Å thick) was used for the DFT calculations.

The oxidation states of Pr in the PBC and PBSCF are +3 (**Supplementary Figure 14, Note 7**). We believe that Pr is not involved in the OER process and always maintain a +3 valence state in our transition metal oxygen-layer exposed slab. Admittedly, Pr may have multiple valences; however, no valid evidence proves that Pr can change valence upon oxygen vacancy formation in double-perovskite $PrBaCo_2O_6$. To the best of our knowledge, it is widely treated as Pr_3 in all DFT calculations of double-perovskite materials[22]. Moreover, it has been widely accepted that the B-site ion is the active site for OER[19], which contributes to the intrinsic OER activity of perovskite catalysts[2]. Importantly, for the double perovskites $(Ln_{0.5}Ba_{0.5})CoO_{3-\delta}$ (Ln = Pr, Sm, Gd and Ho), it was found that the OER activity depends sensitively on the e_g electron filling of the B-site ion[1], not on the property of Ln. Accordingly, it's reasonable to just consider the B-site ions (Co, Fe in PBSCF) as the active sites.

Sr and Fe doping concentrations on Ba and Co sites are 0.5 and 0.25. The in-plane lattice $\sqrt{2} \times \sqrt{2}$ expansion was followed by a 45° degree in-plane rotation along z, ending up with 2 transition metal ions per Co/Fe-O layer with the adsorbate surface coverage ½ ML. The simulation slabs used for the DFT calculation were fully oxidized and relaxed structure. It's reasonable to use such a fully oxidized and relaxed structure for DFT calculation as suggested by Shao-Horn and co-workers[1] that the surface of these oxide catalyst is expected to be fully oxidized in KOH under OER conditions and the intrinsic OER activities of cobalt-based double perovskites and pseudocubic perovskites were found to correlate with the computed O p-band centre relative to the Fermi level of the fully

oxidized and relaxed structure instead of nonstoichiometric double perovskites or stoichiometric perovskite in the constrained structure. The VASP POSCAR files of PBC and PBSCF slabs were attached as **Supplementary Note 8**. For PBSCF, both Co and Fe redox site were examined for OER reactions. The relaxed atomic structures of adsorbed slabs for PBC and PBSCF were shown in **Supplementary Figure 16**.

The PDOS diagrams are shown in **Supplementary Figure 17**. Only significant O *p*-band, Co and Fe *d*-band are shown. Near valence band maximum, dominant O *p*-band (red curves) is observed.

The O *p*-band center was calculated based on the PDOS of $PrBa_{0.5}Sr_{0.5}Co_{1.5}Fe_{0.5}O_6$ by integrating oxygen PDOS over *p*-band using the equation shown below:

$$p - band\ center = \frac{\int E \cdot f(E)dE}{\int f(E)dE} \qquad (6)$$

where E is the electron energy, $f(E)$ is the PDOS value.

The obtained *p*-band center is -1.888 eV, close to that of reported double perovskite family[1].

Supplementary References

1. Grimaud, A., *et al.* Double perovskites as a family of highly active catalysts for oxygen evolution in alkaline solution. *Nat. Commun.* **4**, 2439 (2013).

2. Suntivich, J., May, K. J., Gasteiger, H. A., Goodenough, J. B., Shao-Horn, Y. A perovskite oxide optimized for oxygen evolution catalysis from molecular orbital principles. *Science* **334**, 1383-1385 (2011).

3. Zhu, Y. L., *et al.* $SrNb_{0.1}Co_{0.7}Fe_{0.2}O_{3-\delta}$ perovskite as a next-generation electrocatalyst for oxygen evolution in alkaline solution. *Angew. Chem. Int. Ed.* **54**, 3897-3901 (2015).

4. Moulder, J. F., Sticlke, W. F., Sobol, P. E., Bomben, K. D. Handbook of X-ray Photoelectron Spectroscopy. Chastain, J., Ed.; PerkinElmer Corporation: Eden Prairie, MN,. (1992).

5. Paunovic, N., *et al.* Suppression of inherent ferromagnetism in Pr-doped CeO_2 nanocrystals. *Nanoscale* **4**, 5469-5476 (2012).

6. Borchert, H., *et al.* Electronic and chemical properties of nanostructured cerium dioxide doped with praseodymium. *J. Phys. Chem. B* **109**, 5728-5738 (2005).

7. Kresse, G., Furthmuller, J. Efficient iterative schemes for ab initio total-energy calculations using a plane-wave basis set. *Phys. Rev. B* **54**, 11169-11186 (1996).

8. Perdew, J. P., Burke, K., Ernzerhof, M. Generalized gradient approximation made simple. *Phys. Rev. Lett.* **77**, 3865-3868 (1996).

9. Kresse, G., Joubert, D. From ultrasoft pseudopotentials to the projector augmented-wave method. *Phys. Rev. B* **59**, 1758-1775 (1999).

10. Dudarev, S. L., Botton, G. A., Savrasov, S. Y., Humphreys, C. J., Sutton, A. P. Electron-energy-loss spectra and the structural stability of nickel oxide: An LSDA+U study. *Phys. Rev. B* **57**, 1505-1509 (1998).

11. Ritzmann, A. M., Dieterich, J. M., Carter, E. A. Density functional theory plus U analysis of the electronic structure and defect chemistry of LSCF ($La_{0.5}Sr_{0.5}Co_{0.25}Fe_{0.75}O_{3-\delta}$). *Phys. Chem. Chem. Phys.* **18**, 12260-12269 (2016).

12. Pack, J. D., Monkhorst, H. J. "Special points for Brillonln-zone integrations"—a reply. *Phys. Rev. B* **16**, 1748-1749 (1977).

13. Pulay, P. Convergence acceleration of iterative sequences - the case of SCF iteration. *Chem. Phys. Lett.* **73**, 393-398 (1980).

14. Arias, T. A., Payne, M. C., Joannopoulos, J. D. Abinitio molecular-dynamics-analytically

continued energy functionals and insights into iterative solutions. *Phys. Rev. Lett.* **69**, 1077-1080 (1992).

15. Norskov, J. K., *et al.* Origin of the overpotential for oxygen reduction at a fuel-cell cathode. *J. Phys. Chem. B* **108**, 17886-17892 (2004).

16. *NIST JANAF Thermochemical Tables*. http://kinetics.nist.gov/janaf/.

17. Noh, J. S., Schwarz, J. A. Estimation of the point of zero charge of simple oxides by mass titration. *J. Colloid Interf. Sci* **130**, 157-164 (1989).

18. Pechenyuk, S. I. The use of the pH at the point of zero charge for characterizing the properties of oxide hydroxides. *Russ. Chem. B+.* **48**, 1017-1023 (1999).

19. Hong, W. T., *et al.* Toward the rational design of non-precious transition metal oxides for oxygen electrocatalysis. *Energy Environ. Sci.* **8**, 1404-1427 (2015).

20. Kushima, A., Yip, S., Yildiz, B. Competing strain effects in reactivity of $LaCoO_3$ with oxygen. *Phys. Rev. B* **82**, 115435 (2010).

21. Wang, Y., Cheng, H. P. Oxygen reduction activity on perovskite oxide surfaces: a comparative first-principles study of $LaMnO_3$, $LaFeO_3$, and $LaCrO_3$. *J. Phys. Chem. C* **117**, 2106-2112 (2013).

22. Choi, S., *et al.* Highly efficient and robust cathode materials for low-temperature solid oxide fuel cells: $PrBa_{0.5}Sr_{0.5}Co_{2-x}Fe_xO_{5+\delta}$. *Sci. Rep.* **3**, 2426 (2013).
